# A unique combination of natural fatty acids from *Hermetia illucens* fly larvae fat effectively combats virulence factors and biofilms of MDR hypervirulent mucoviscus *Klebsiella pneumoniae* strains by increasing Lewis acid–base/van der Waals interactions in bacterial wall membranes

**DOI:** 10.3389/fcimb.2024.1408179

**Published:** 2024-07-25

**Authors:** Heakal Mohamed, Elena Marusich, Mikhail Divashuk, Sergey Leonov

**Affiliations:** ^1^ Agricultural Research Center (ARC), Plant Protection Research Institute (PPRI), Giza, Egypt; ^2^ The Laboratory of Personalized Chemoradiotherapy, Institute of Future Biophysics, Moscow, Russia; ^3^ All-Russia Research Institute of Agricultural Biotechnology Kurchatov Genomic Center - VNIISB, Moscow, Russia; ^4^ Institute of Cell Biophysics, Russian Academy of Sciences, Moscow, Russia

**Keywords:** *Hermetia illucens*, fatty acids, hvKp, CR-KP, virulence factors, biofilms, Lewis acid-base, van der Waals interactions

## Abstract

**Introduction:**

Hypervirulent *Klebsiella pneumoniae* (hvKp) and carbapenem-resistant *K. pneumoniae* (CR-Kp) are rapidly emerging as opportunistic pathogens that have a global impact leading to a significant increase in mortality rates among clinical patients. Anti-virulence strategies that target bacterial behavior, such as adhesion and biofilm formation, have been proposed as alternatives to biocidal antibiotic treatments to reduce the rapid emergence of bacterial resistance. The main objective of this study was to examine the efficacy of fatty acid-enriched extract (AWME3) derived from the fat of Black Soldier Fly larvae (*Hermetia illucens*) in fighting against biofilms of multi-drug resistant (MDR) and highly virulent *Klebsiella pneumoniae* (hvKp) pathogens. Additionally, the study also aimed to investigate the potential mechanisms underlying this effect.

**Methods:**

Crystal violet (CV) and ethidium bromide (EtBr) assays show how AWME3 affects the formation of mixed and mature biofilms by the KP ATCC BAA-2473, KPi1627, and KPM9 strains. AWME3 has shown exceptional efficacy in combating the hypermucoviscosity (HMV) virulent factors of KPi1627 and KPM9 strains when tested using the string assay. The rudimentary motility of MDR KPM9 and KP ATCC BAA-2473 strains was detected through swimming, swarming, and twitching assays. The cell wall membrane disturbances induced by AWME3 were detected by light and scanning electron microscopy and further validated by an increase in the bacterial cell wall permeability and Lewis acid-base/van der Waals characteristics of *K. pneumoniae* strains tested by MATS (microbial adhesion to solvents) method.

**Results:**

After being exposed to 0.5 MIC (0.125 mg/ml) of AWME3, a significant reduction in the rudimentary motility of MDR KPM9 and KP ATCC BAA-2473 strains, whereas the treated bacterial strains exhibited motility between 4.23 ± 0.25 and 4.47 ± 0.25 mm, while the non-treated control groups showed significantly higher motility ranging from 8.5 ± 0.5 to 10.5 ± 0.5 mm.

**Conclusion:**

In conclusion, this study demonstrates the exceptional capability of the natural AWME3 extract enriched with a unique combination of fatty acids to effectively eliminate the biofilms formed by the highly drug-resistant and highly virulent *K. pneumoniae* (hvKp) pathogens. Our results highlight the opportunity to control and minimize the rapid emergence of bacterial resistance through the treatment using AWME3 of biofilm-associated infections caused by hvKp and CRKp pathogens.

## Graphical Abstract

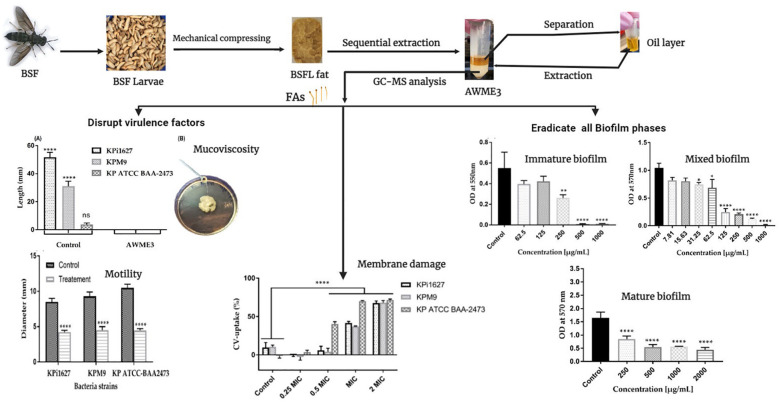

## 1 Introduction

Misuse and overuse of antibiotics have led to the emergence of drug-resistant bacteria, which is a major threat to global health. In fact, WHO has declared that “Antimicrobial resistance (AMR)” is one of the top global public health and development threats. Multidrug resistance (MDR) has increased all over the world, which threatens public health. Several recent investigations reported the emergence of multidrug-resistant bacterial pathogens from different origins that increase the necessity of new potent and safe alternatives for antibiotics. Besides, the routine application of antimicrobial susceptibility testing detects the antibiotic of choice as well as the screening of emerging MDR (Algammal et al., 2019; Kareem et al., 2021; Elbehiry et al., 2022; Shafiq et al., 2022; Algammal et al., 2022a, b). AMR bacteria caused around 1.27 million deaths globally in 2019 and contributed to 4.95 million deaths (Murray et al., 2022). By 2050, more people are expected to die from antibiotic-resistant infections than from cancer (O’Neill, 2014). We need to urgently find effective alternatives to deal with this crisis. There is now more research being done on different alternative ways to fight bacteria, whereas these approaches are developed in different stages, including the use of novel antibiotics, phage therapy, antimicrobial peptides, nanoparticles, and anti-virulence, which are considered as one such promising approach for overcoming bacterial resistance (Alaoui Mdarhri et al., 2022). One of these approaches is the anti-virulence approach, which is promising to provide novel antimicrobial therapies predicted to be superior to conventional antibiotics (Allen et al., 2014; Totsika, 2017). Anti-virulence strategies that target bacterial behavior, such as adhesion and biofilm formation, are anticipated to apply minimal selective pressure, which aims to reduce virulence and less likely to induce drug resistance. These strategies mainly focus on neutralizing virulence factors and declining bacterial infection without direct killing or elimination of the bacteria. Accordingly, there is less selective pressure on bacterial survival, thus less likely to induce drug resistance (DIckey et al., 2017; Maura et al., 2016). There are a variety of virulence factors in hvKP, including virulence genes, virulence plasmids, capsular polysaccharide, siderophore, lipopolysaccharide, and fimbriae, which play a crucial role in bacterial infection and resistance (Liao et al., 2024).

*Klebsiella pneumoniae* is an important opportunistic human pathogen commonly involved in hospital-acquired infections (Lam et al., 2018; Piepenbrock et al., 2020). The hvKp strain is particularly virulent causing invasive and metastatic infections even in young and healthy individuals. Moreover, hvKp is easily transmitted leading to infections in multiple sites such as the thorax, abdomen, central nervous system, eyes, and genitourinary tract (Sellick and Russo, 2018). Most alarmingly, CR-Kp has emerged and caused severe and fatal infections in healthcare settings (Tang et al., 2020). *K. pneumoniae* causes several infections via gene or plasmid horizontal transfer (Wyres and Holt, 2018).

Most microorganisms in a biofilm grow slowly, exhibit downregulated virulence, and are distributed heterogeneously. Biofilms are harder to be killed with antibiotics than individual cells. They can also avoid being removed by the immune system (Jefferson, 2004; Hu et al., 2012; Jennings et al., 2015). Biofilm-related infections cover a range of conditions, from infections related to medical devices, like prosthetic joints, to infections affecting native tissues, like chronic osteomyelitis and cystic fibrosis.

Biofilms are intricate communities of microorganisms that are surrounded by an extracellular matrix made up of proteins, extracellular DNA (eDNA), lipids, and exopolysaccharides (Jennings et al., 2015). Bacterial biofilms grow in a way that has many benefits, including the bacteria staying in a small environment as long as the conditions are good. In biofilms, bacteria make up less than 10% of their dry mass, while the matrix can make up over 90%. This matrix is composed of various types of biopolymers collectively known as extracellular polymeric substances (EPS). The EPS, produced by the organisms themselves, enables bacterial cells to live in proximity and interact. This behavior is significantly different from their planktonic counterparts (Hu et al., 2012).

The hvKp strains can form biofilms and remain persistent inside the biofilms thereby enhancing virulence and invasive capacity of infection through colonization in the respiratory, gastrointestinal, and urinary tracts. During biofilm formation, exopolysaccharides are produced by bacterial cells forming a matrix around the cells to protect them from the harsh environmental conditions and exposure to bioactive agents. The current treatment for these infections involves removing the infected medical device and cleaning the affected tissue with antibiotics. However, treating these infections is still difficult. Promising research is being done on new anti-biofilm agents like quorum-sensing inhibitors, biofilm matrix-degrading enzymes, and antimicrobial peptides. These potential candidates hold the key to overcoming the hurdles posed by biofilm-related infections.

Recently, we demonstrated that fatty acid (FA)-enriched fractions of *Hermetia illucens* (HI) (Black soldier fly) larvae oil possess bactericidal activity against hypervirulent mucoviscous *K. pneumoniae* strains, actual phytopathogens, and multi-drug resistant (MDR) pathogenic fish bacteria (Marusich et al., 2020; Mohamed et al., 2021, 2022). In particular, the third acidic water–methanol extract (AWME3) demonstrated an exceptional ability to eliminate MDR and XDR *K. pneumoniae* strains at low doses. *Hermetia illucens* (HI) is a remarkable insect species because its larvae have the ability to produce FAs through biosynthesis pathways rather than solely relying on bioaccumulation from their diet. This makes them highly promising compared to other insects. Larvae are full of natural substances that can kill bacteria and could be used to treat serious infections caused by antibiotic-resistant bacteria. HI larvae contain 15%–49% fat providing a rich lipid source (Li et al., 2016).

In the present study, we further explore the anti-biofilm and anti-virulence properties of AWME3 against biofilms formed by *K. pneumoniae* strains isolated from Russian hospitals between 2011 and 2016, including mucoviscous KPM9, hyper-mucoviscous KPi1627, and the standard non-mucoid NDM-1 carbapenemase-resistant KP ATCC BAA-2473 strains. We also investigate how AWME3 activity affects bacterial membrane permeability and the Lewis acid–base/van der Waals properties. These changes represent the mechanistic key to understanding superior AWME3’s sub-MIC activity against virulence factors, such as mucoviscosity and rudimentary motility, and different types of biofilms formed by the three tested *K. pneumoniae* strains.

## 2 Materials and methods

### 2.1 Chemicals and media

Different chemicals used in this study, including acetic acid (CH_3_COOH), ethanol (C_2_H_5_OH), hexane (C_6_H_14_), chloroform (CHCl_3_), ethyl acetate (C_4_H_8_O_2_), and toluene (C_7_H_8_), were purchased from Thermo Fisher Scientific, Waltham, USA. Crystal violet, propidium iodide, ethidium bromide, glutaraldehyde, phosphate buffer saline (PBS) were purchased from Sigma-Aldrich, St. Louis, USA. Methanol (CH_3_OH) and hydrochloric acid (HCl), purchased from Sigma-Aldrich, St. Louis, USA, and Milli-Q H_2_O were mixed together with the intended ratio for extraction procedure. Luria–Bertani (LB) broth and Mueller–Hinton (MH) broth (Sigma-Aldrich, St. Louis, USA) were used to culture bacteria in liquid media. LB and MH agar (Sigma-Aldrich, St. Louis, USA) were used to culture bacteria on solid media. Tryptone soy agar (Oxoid, Basingstoke, Hampshire, United Kingdom) was used to determine twitching motility of bacteria. Peptone, tryptone, and NaCl were purchased from Sigma-Aldrich, St. Louis, USA, while yeast extract was purchased from Difco, USA, and used to prepare the culture media to validate the bacterial motility.

### 2.2 Bacterial strains and growth conditions

Environmental isolate *K. pneumoniae* KPM9 and clinical isolate *K. pneumoniae* KPi1627 strains were obtained from the State Collection of Pathogenic Microorganisms and Cell Cultures (SCPM, Obolensk, Russia). *K. pneumoniae* ATCC BAA-2473 laboratory strain was purchased from ATCC (American Type Culture Collection, United States). All tested bacteria strains were identified according to Lev et al. (2018). *K. pneumoniae* KPi1627 strain was isolated from a clinical sample (trachea) at Moscow Infectious Hospital No. 1 in 2014, while *K. pneumoniae* KPM9 strain was isolated from the environment (fresh-water) in the Krasnodar Region of Russia in 2011 (Lev et al., 2018) and collected in the Burdenko Neurosurgery Institution.

The identification and detection of bacterial strains were confirmed using a Vitek-2 Compact instrument with a VITEK^®^ 2 Gram-negative (GN) ID card (SKU number 21341; BioMérieux, Paris, France) and a MALDI-TOF Biotyper (Bruker Daltonics, Bremen, Germany) instrument, which is capable of distinguishing among *Klebsiella oxytoca*, *K. pneumoniae* subsp. *ozaenae*, *K. pneumoniae* subsp. *pneumoniae*, *K. pneumoniae* subsp. rhinoscleromatis, and *K. variicola*. After that, identified *K. pneumoniae* strains were stored in 15% glycerol and kept at −80°C. A single colony from each strain was inoculated in 10 ml of the LB broth and incubated overnight at 37°C by shaking at 210 rpm/min. The overnight culture was adjusted to half of the McFarland standard (1 × 10^8^ CFU/ml) to be used in biofilms assays under static conditions.

### 2.3 Extraction method

Acidic water–methanol extract (AWME3) was isolated from live Black Soldier Fly (*H. illucens*) larvae, 15 days old, brownish color, wheat fed, and provided by the NordTechSad, LLC company (Arkhangelsk, Russia). The *H. illucens* larvae fat was extracted according to Mohamed et al. (2022). Briefly, 3 g of larvae fat was subjected to sequential extraction using water (Milli Q quality), methanol (99.9%, HPLC grade), and hydrochloric acid (37%) with a ratio of 90:9:1, v/v/v. AWME3 was selected for our experiments in this study because of its highest activity among other extracts against *Aeromonas* sp (Mohamed et al., 2021).

### 2.4 Membrane permeability of *K. pneumoniae* strains

The impact of AWME3 on the alteration of the membrane permeability of all *K. pneumoniae* strains was determined using crystal violet (CV) uptake assay, in which stain is passed through the cell membrane (Hobby et al., 2019). After incubation in LB medium at 37°C overnight, *K. pneumoniae* strains were harvested and washed with PBS, pH 7.4, three times. The pellets were resuspended in PBS and mixed with AWME3 at a concentration from ¼ to 2 MIC for 4 h. Bacterial cells were incubated with 1% CV in the dark for 15 min. After centrifugation, the absorbance of the supernatant was determined by measuring the OD_570_ nm using CLARIOstar^®^ *Plus* multimodal plate reader (BMG Labtech, Ortenberg, Germany). The absorbance of CV was considered as 100%. The crystal violet uptake was calculated using the following formula: % of uptake = (OD of the sample)/(OD of the crystal violet solution) × 100.

### 2.5 Virulence factor analysis (motility assays)

#### 2.5.1 Swarming motility

*K. pneumoniae* strains were grown in LB agar (Sigma, USA) with or without sub-MIC of AWME3 for 24 h at 30°C. Then, swarming agar plates containing 1% glucose (Sigma-Aldrich, St. Louis, USA), 0.5% peptone (Sigma-Aldrich, St. Louis, USA), 0.2% yeast extract (Oxoid, Basingstoke, Hampshire, UK), and 0.5% agar (Sigma-Aldrich, St. Louis, USA) were equilibrated to room temperature and inoculated at the center with 10 µl of each strain of *K. pneumoniae* suspension containing 10^8^ CFU/ml in the presence and absence (control) of sub-MIC of AWME3. Plates were incubated without inversion for 24 h at 30°C (Saeki et al., 2021).

#### 2.5.2 Swimming motility

All three *K. pneumoniae* strains were seeded on LB agar (Sigma-Aldrich, St. Louis, USA) and incubated at 37°C for 24 h. Then, one colony of each isolate was inoculated in the presence and absence (control) of ½ MIC AWME3 on the surface of swimming agar plates, containing 1.0% tryptone (Oxoid, Basingstoke, Hampshire, UK), 0.5% sodium chloride (Sigma-Aldrich, St. Louis, USA), and 0.3% agar (Difco, USA) and previously equilibrated to room temperature. Plates were incubated without inversion for 24 h at 30°C (Saeki et al., 2021).

#### 2.5.3 Twitching motility

All *K. pneumoniae* bacteria strains were seeded on LB agar (Sigma-Aldrich, St. Louis, USA) and incubated at 37°C for 24 h. Then, one colony of each isolate was inoculated in the presence and absence (control) of ½ MIC AWME3 to the bottom of twitching agar plates containing 1.0% tryptone (Oxoid, Basingstoke, Hampshire, UK), 0.5% yeast extract (Oxoid, UK), 1.0% sodium chloride (Sigma, USA), and 1.0% agar (BD Difco, New Jersey, USA). Plates were inverted and incubated at 37°C for 24 h. Subsequently, the agar was carefully removed, and the motility zone was measured to the nearest millimeter after staining with 2% crystal violet (Sigma-Aldrich, St. Louis, USA) for 2 h (Hu et al., 2012). As a negative control, each strain was inoculated in tryptone soy agar (BD Difco, New Jersey, USA) under the same conditions.

### 2.6 Minimal inhibitory biofilm concentration test

The MIBC assay was conducted *via* the microdilution assay described by Cepas et al. (2019) with some modifications. For all *K. pneumoniae* strains, 100 µl of twofold dilutions of AWME3 in LB broth media with 1-, 0.5-, 0.25-, 0.125-, 0.063-, 0.032-, 0.016-, and 0.08-mg/ml concentrations in LB broth was inoculated with 100 µl of bacterial suspension 1 × 10^6^ CFU/ml and incubated for 24 h at 37°C without shaking. A negative control (culture medium without inoculum) and a positive control (culture medium with inoculum) were included in each 96-well plate. All plates were covered with adhesive film to avoid evaporation. After incubation, the unattached cells were carefully removed and washed twice with PBS and dried at 60°C for 20 min. Attached biofilms were stained with 125 µl of 1% (v/v) of CV and incubated for 10 min at room temperature. Afterward, the CV was completely removed, washed with PBS, and dried at 65°C for 60 min. The plates were rinsed with d.H_2_O and dried, followed by the addition of 125 µl of 30% acetic acid to dissolve the biofilm-bound dye. Optical density was measured at 570 nm (OD_570_) using a CLARIOstar^®^ *Plus* multimodal plate reader (BMG Labtech, Ortenberg, Germany). The MBIC was defined as the lowest concentration of AWME3, which resulted in a threefold decrease in OD_570_, in comparison with the positive growth-control value (only bacteria). Additionally, MIBC of AWME3 and positive control (Dox) was determined in 96-well plates and identified as the lowest concentration, which inhibits biofilm growth of *K. pneumoniae* strains.

### 2.7 Minimum eradication biofilm concentration test

The minimum eradication biofilm concentration (MEBC) was determined according to Balkrishna et al. (2021) with minor changes. The MEBC was determined based on the MIBC test, where different concentrations of AWME3, in the range 0.08–1.0 mg/ml, were inoculated with fixed 1 × 10^6^-CFU/ml concentration of each *K. pneumoniae* strain in sterile LB broth and incubated at 37°C for 24 h. Subsequently, an aliquot of 30 μl of each of the MIBC, 2 MIBC, and 4 MIBC was scraped and spread on sterile MH agar plates then incubated for 48 h at 37°C. The lowest concentration of AWME3, which prevented bacterial growth, was identified as the MEBC. Likewise, doxycycline was used as a positive control, and MEBC was determined by the same manner.

### 2.8 Testing of AWME3 action against mature biofilms

The biofilm disruption assay was performed in a 96-well polystyrene plate (TPP, Trasadingen Switzerland) following the procedure (Chmielewska et al., 2020; Wijesinghe et al., 2021) with minor modifications. The bacteria were grown in microtiter plates for 72 h at 37°C to form mature biofilms in the wells. Then, media was discarded gently, and wells were washed using PBS buffer to remove loosely adhered cells. Freshly prepared LB broth was added to each well, and then AWME3 was added to give final concentrations of 0.25, 0.500, 1.0, and 2.0 mg/ml. The plate was incubated for the next 24 h under static conditions at 37°C. The wells of the plates were washed by sterile PBS to remove the planktonic cells followed by staining with 0.1% CV solution in water for 30 min. The stain was removed and gently washed with d.H_2_O, dried at 60°C for 60 min, and the remaining biofilm-bound dye was dissolved using 30% acetic acid. The OD570 was recorded using a CLARIOstar microplate reader, and the percent of biofilm disruption was calculated with respect to the control group. The MEBC of AWME3 and doxycycline was determined by counting formed colonies (CFU). Briefly, adhered treated biofilms were completely scraped and serially diluted in PBS. Of prepared dilutions, 30 µl was spread on MH agar medium separately, then incubated at 37°C for 48 h; MEBC identified as the lowest concentration of AWME3 or doxycycline, which was able to eliminate the bacterial biofilm growth.

### 2.9 Fluorescence microscopy

Biofilm architecture, in the absence and presence of AWME3 antimicrobial, was evaluated using the fluorescence microscopy protocol of Sateriale et al. (2020) and described in the **Supplementary Methods** in light microscopy examination with some changes. After fixing wells with 90% ethanol for 15 min and completely drying at 30°C, the biofilms were stained with 1 mM propidium iodide (PI) for 15 min at room temperature. The excess dye was washed with d.H_2_O. Finally, biofilms were observed with a fluorescent microscope (Life technologies, Bothell, WA, USA) equipped with a digital camera. Digital images were acquired using a ×4 objective at PI excitation/emission wavelength of 543/617 nm. All obtained images were analyzed using Fiji Image J software (National Institutes of Health, Bethesda, USA) to obtain the mean fluorescence intensities from digital fluorescent images of biofilms.

### 2.10 Scanning electron microscopy

Treated and untreated biofilms of *K. pneumoniae* ATCC BAA-2473 strain were examined using a scanning electron microscope (SEM) according to Ceruso et al. (2020) with minor modifications. Briefly, the *K. pneumoniae* cells were cultured and grown on 1-cm^2^ cover glass (Thermo Fisher Scientific, Waltham, USA) in a six-well plate (TPP, Switzerland) for 6 h as mentioned above. Next, 0.0-, 0.125-, 0.250-, and 0.500-mg/mL concentrations of AWME3 were added to the formed biofilm in a six-well microtiter plate, then incubated for 24 h at 37°C without shaking. Further, the planktonic cells were removed by washing with PBS, pH7.4, three times. All adhered biofilms on the surface of the glass coverslips were fixed with 2.5% glutaraldehyde, pH 7.2, overnight at 4°C, washed three times in the rinsing buffer PBS at 4°C for 15 min, and then dehydrated by ethanol solutions in following concentrations: 30%, 50%, 70%, 80%, 90%, and 95%. All dehydrated samples were visualized under SEM (TESCAN, Kohoutovice, Czech Republic).

### 2.11 Statistical analysis

Statistical analysis was conducted, and graphs were generated using the software GraphPad Prism 7 (GraphPad Software Inc., San Diego, CA, United States). All experiments were performed in triplicate validating the statistical significance by one-way ANOVA test with Dunnett’s multiple comparison test and two-way ANOVA test with Dunnett’s, Tukey’s, and Sidak’s corrections, and the statistical significance level was p < 0.05.

## 3 Results

Our study focused on exploring the anti-biofilm and anti-virulence properties of AWME3 against biofilms formed by *K. pneumoniae* strains isolated from Russian hospitals between 2011 and 2016. This included mucoviscous KPM9, hyper-mucoviscous KPi1627, and the standard non-mucoid NDM-1 carbapenemase-resistant KP ATCC BAA-2473 strains.

### 3.1 Phenotypic characteristics of the tested *K. pneumoniae* strains

*K. pneumoniae* KPi1627 and *K. pneumoniae* KPM9 strains demonstrated multidrug-resistant phenotypes to more than three different classes of antibiotics (Magiorakos et al., 2012), while *K. pneumoniae* ATCC BAA-2473 was classified as extensive drug resistant (XDR) (Mohamed et al., 2022). In addition, *K. pneumoniae* KPi1627 and *K. pneumoniae* KPM9 displayed high hypermucoviscosity after string assay, while *K. pneumoniae* ATCC BAA-2473 was negative to the same test.

### 3.2 AWME3 impact on MDR *K. pneumoniae* strains grown under biofilm vs. planktonic bacterial mode

To enhance the diagnosis, treatment, and prevention of infections, it is crucial to differentiate between acute infections caused by the independent growth of individual microorganisms (the so-called “planktonic” growth) and biofilm infections, which involve clusters of microbial cells (Mirzaei et al., 2024). The minimum inhibitory concentration (MIC) is the lowest concentration of antibiotics that stops visible bacterial growth. The minimum bactericidal concentration (MBC) is the lowest concentration needed to kill the bacteria. Diagnostic laboratories use MICs primarily to confirm the presence of resistance. MIC and MBC are determined based on planktonic cells, while the minimum biofilm eradication concentration (MEBC) indicates the lowest antibiotic concentration needed to eliminate the biofilm.

In a previous study, we determined MIC and MBC of AWME3 extract against planktonic bacterial cells of three *K. pneumoniae* isolates, including KPi1627, KPM9, and KP ATCC BAA-2473 (Mohamed et al., 2022). The MIC and MBC were recorded as 250 µg/ml under planktonic growth conditions in these experiments. In the present study, we identified the minimum inhibition biofilm concentration (MIBC) and MEBC for the same strains as 500 µg/ml, when they were exposed to static conditions after being adherent to polystyrene microtiter plates with the exceptional resilience (**Table 1**). Doxycycline (Dox) used as the positive control displayed remarkable MIC values of 6.25, 3.12, and 12.5 µg/ml against *K. pneumoniae* KPi1627, *K. pneumoniae* KPM9, and *K. pneumoniae* ATCC BAA-2473, respectively. At the same time, Dox exhibited MEBC values exceeding 50 µg/ml for all three strains.

**Table 1:** Antibacterial activity of AWME3 against *K. pneumoniae* strains grown under biofilm *vs.* planktonic bacterial mode.

Incubation condition	Activity		Concentration (μg/ml)		
			KP ATCC BAA-2473	KPM9	KPi 1627
Under shaking (planktonic cells)	AWME3	MIC	250	250	250
		MBC	250	250	250
Static (adherent cells)		MIBC	500	500	500
		MEBC	500	500	500
Under shaking (planktonic cells)	Dox	MIC	6.25	3.12	1.56
		MBC	50	12.5	6.25
Static (adherent cells)		MIBC	3.13	6.25	12.5
		MEBC	>50	>50	>50

These results demonstrate a significant reduction of sensitivity to AWME3 treatment by all hvKp-tested strains when they were grown in biofilm mode compared to that of planktonic cell growth. Moreover, the AWME3 extract demonstrated bactericidal activity, comparable to the standard antibiotic (Dox) against all biofilms formed by *K. pneumoniae* strains (**Table 1**).

### 3.3 AWME3 impact on preformed biofilms of MDR *K. pneumoniae* strains

Biofilms are constructed as bacterial colonies formed in multiple layers on biotic or abiotic material that facilitates bacterial survival and persistence against harmful conditions, as well as contributes to virulence during infection. We performed biofilm formation assay using LB broth medium in a 96-well microtiter plate. The biofilms grown for 24 h were assessed by staining with crystal violet (CV). Crystal violet, an aniline dye, represents the initial stain employed in the process of Gram staining. When cells are exposed to a 95% ethanol or acetone solution, they create a vivid and eye-catching purple color after interacting with the crystal violet pigment. The intensity of CV staining in *K. pneumoniae*, Gram-negative bacteria, reveals the extent of the thin peptidoglycan layer covered by lipopolysaccharides and lipoproteins, proteins, and DNA, which form a biofilm on the plastic surface.

**Figure 1:**
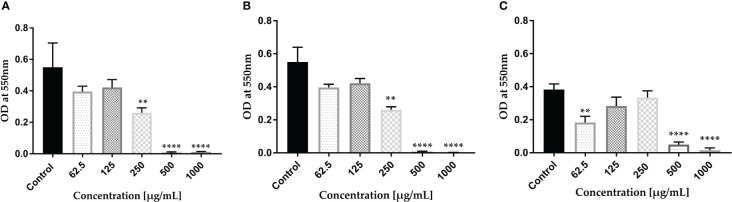
AWME3 effect against preformed biofilms of *K pneumoniae* **(A)** KPi1627, **(B)** KPM9, and **(C)** KP ATCC BAA-2473 strains. The experiments were performed in triplicate with independent cultures, and statistical significance was examined by the one-way ANOVA test with Dunnett’s multiple comparison test. Results are indicated as means ± STDs. Asterisks indicate statistical significance (**p = 0.001, ****p < 0.0001).

The intensity of biofilm staining was accurately measured by meticulously dissolving the dye in a 30% acetic acid and water mixture. Our results of the CV dye biofilm staining demonstrate that KPi1627, KPM9, and KP ATCC BAA-2473 strains form strong biofilms (**Figure 1**). Significant inhibition of biofilm formation (p < 0.0001) by all *K. pneumoniae* strains was observed at concentrations MIBC (0.5 mg/ml) and 2 MIBC (1.0 mg/ml). KPi1627 and KPM9 biofilms were significantly reduced (p = 0.001) at 0.5 MIBC (0.25 mg/mL) of AWME3 (**Figures 1A, B**). Of note, the KP ATCC B AA-2473 strain biofilm was significantly reduced (p = 0.007) after exposition to 0.125 MIBC (0.0625 mg/ml) of AWME3 (**Figure 1C**).

### 3.4 AWME3 effect on mixed biofilms of MDR *K. pneumoniae* strains

The tested opportunistic MDR *K. pneumoniae* pathogens frequently form mixed biofilms that can lead to nosocomial infections in healthy individuals. We wanted to test how well AWME3 works against resistant strains and if it can remove the strong biofilms created by these strains. Mixed biofilm established by equal volumes (1:1:1) of the three different strains was the strongest one (p = 0.003) compared to biofilms formed by each *K. pneumoniae* strains alone (**Supplementary Figure S1**). On the other hand, KP ATCC BAA-2437 was the lowest (p = 0.0005) in biofilm formation capacity, compared to mixed biofilm (**Supplementary Figure S1**). The results depicted in **Supplementary Figure S2** provide compelling evidence of the dose-related eradication of mixed biofilms formed by three strains of *K. pneumoniae*. Notably, when exposed to a concentration of 2 MIBC (1,000 µg/ml) of AWME3, the mixed biofilms consisting of KPi1627, KPM9, and KP ATCC BAA-2473 were completely eradicated (p < 0.0001) (see **Supplementary Figure S2**). Concentrations of 0.25 MIBC (125 µg/ml), 0.5 MIBC (250 µg/ml), MIBC (500 µg/ml), and 2 MIC (1,000 µg/ml) exhibit a remarkable (p < 0.0001) inhibition of the mixed biofilms resulting in 76.3%, 80.01%, 88.2%, and 98.56% reductions, respectively. In addition, our AWME3 extract effectively reduces mixed biofilms to 28.3% and 33.97% at low concentrations of 1/16 MIBC (62.5 µg/ml) and 1/32 MIBC (31.25 µg/ml), respectively, against mixed hvKp strains (**Supplementary Figure S2**) with significant reductions (p = 0.010, p = 0.038).

Our results have conclusively demonstrated that the AWME3 extract derived from HI larvae fat possesses remarkable antimicrobial properties against both single and mixed biofilms formed by various MDR strains of *K. pneumoniae*, such as KPi1627, KPM9, and KP ATCC BAA-2473.

### 3.5 AWME3 disrupts mature biofilms established by MDR *K. pneumoniae* strains

Considering the dynamic growth of biofilms and their greater tolerance to antibiotics, we explored in our study the biofilm growth during 72 h first, and then subjected them to antibiotic challenge. We used light, fluorescence, and scanning electron microscopy as direct microscopic methods to gather evident information about the effect of AWME3 on treated biofilms.

Through the light microscopy technique, we investigated the effect of AWME3 at 0.5 MIBC (0.25 mg/ml), MIBC (0.5 mg/ml), and 2 MIBC (1 mg/ml) on mature biofilm formation on glass coverslips using the CV assay. We found that the control group of bacteria without treatments revealed a remarkable sight. A dense and intricately woven mat of biofilms emerged resembling a heavy knit fabric. These biofilms displayed multiple layers, clearly intact, and adorned the uneven surfaces. Additionally, intriguing clusters of cells gave rise to darkened areas adding to the captivating spectacle. The EPS creates a vast, intertwined network that serves as a matrix for connected threads, effectively shielding the biofilm from a variety of hazardous conditions (**Supplementary Figure S3A**). AWME3 exposures to a sub-MIC (0.25 mg/mL) led to a remarkable decrease in cell count, formation of fragile mats, degradation of clusters, and a noticeable absence of cell aggregation (**Supplementary Figure S3B**). The highest concentration of AWME3 at 2 MIBC (1.0 mg/ml) successfully prevented the formation of mature biofilm in *K. pneumoniae* KP ATCC BAA-2473 leading to the complete absence of cell clusters or aggregates. Furthermore, numerous vacant spaces were observed, and the bacterial cells appeared minimal (**Supplementary Figure S3D**). Notably, there was no bacterial growth after culturing the 30 µl of scraped biofilm on MH agar plates incubated for 24 h, compared to standard antibiotic (Dox), which could not inhibit the mature biofilms at 4 mg/ml (data not shown).

**Figure 2:**
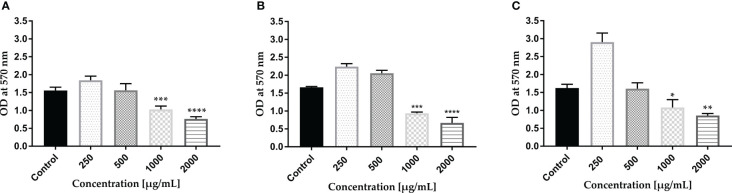
Effect of AWME3 on mature biofilms established by *K pneumoniae* strains **(A)** KPi1627, **(B)** KPM9, and **(C)** KP ATCC BAA-2473. The remaining biofilm mass was stained using CV staining and quantified at 570 nm. Results are the average of three independent experiments ± STDs. The statistical significance was calculated using analysis variance (one-way ANOVA) test with Dunnett’s multiple comparison test. Asterisks indicate statistical significance ****p < 0.0001.

Mature biofilms were also quantified using the CV staining assay in 96-well microplates. The graph in **Figure 2** clearly illustrates that at concentrations of 1,000 and 2,000 µg/ml of AWME3, the biofilms created by *K. pneumoniae* KPi1627, KPM9, and KP ATCC BAA-2473 strains were impressively and significantly disrupted (*p < 0.01–****p < 0.0001).

The mature biofilms formed by the *K. pneumoniae* ATCC BAA-2473 strain clearly exhibited the highest susceptibility to AWME3, with a biofilm reduction of 63.1% when treated with 0.25 mg/ml (**Figure 2C**).

The propidium iodide (PI) staining method is extensively utilized and endorsed in biofilm research giving valuable information regarding eDNA release and degradation that affects biofilm maturation. PI, which can only traverse compromised bacterial membranes, is regarded as an indicator of the integrity of the membrane. Staining based on intact membrane impermeable DNA-binding stains like PI is occasionally used even while specifically studying eDNA (Mann et al., 2009).

**Figure 3:**
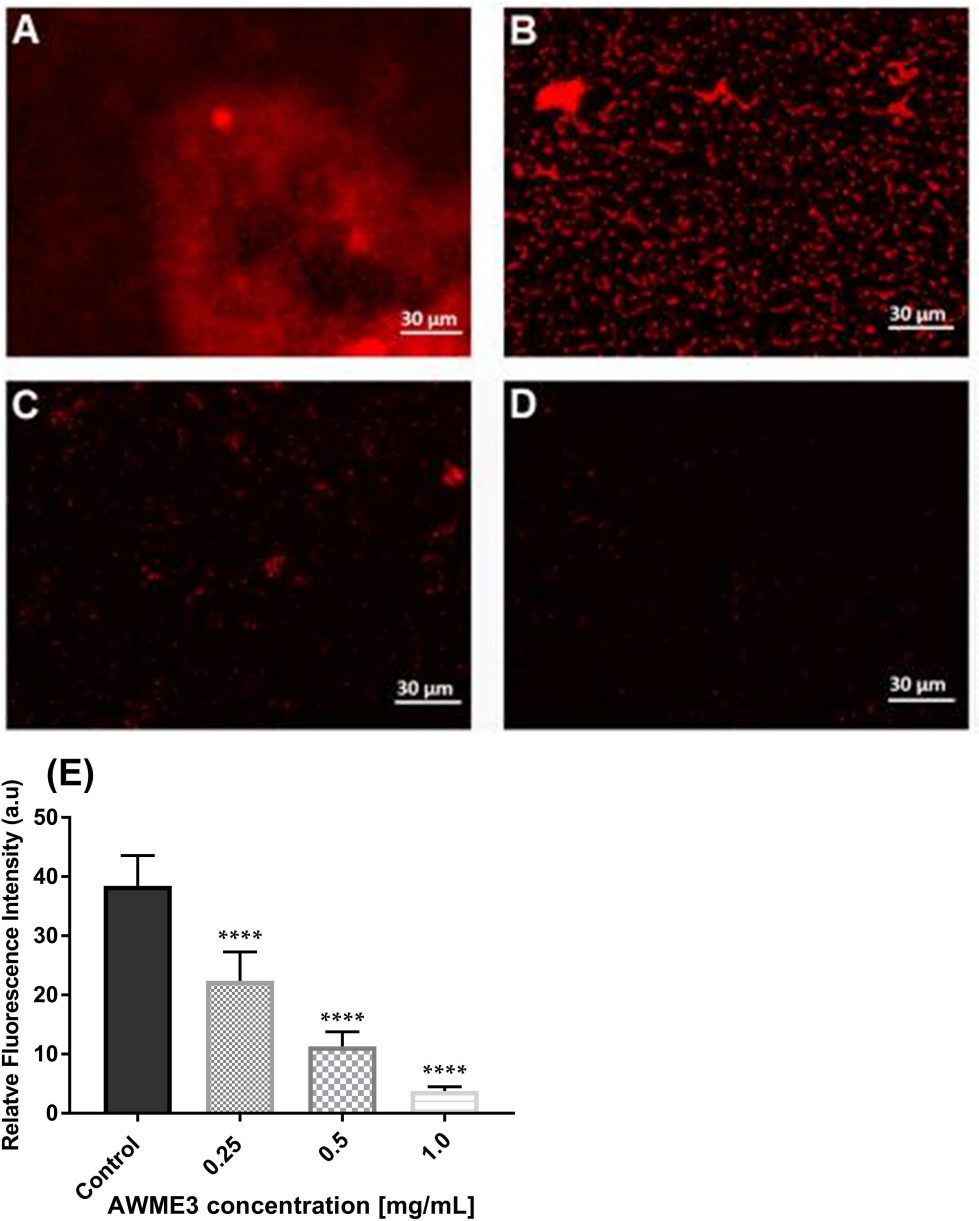
Concentration-related effect of AWME3 on the deposition of extracellular nucleic acids (eNAs) in *K pneumoniae* biofilms. Fluorescence microscopy representative images of **(A)** untreated cell (control) biofilm established by KP ATCC BAA-2473 strain and the same bacteria treated with **(B)** 0.5 MIBC (0.25 mg/ml), **(C)** MIBC (0.5 mg/ml), and **(D)** 2 MIC (1.0 mg/ml) of AWME3. PI staining used to stain eNAs of the biofilm. Relative fluorescence intensity of biofilm structures of the KP ATCC BAA-2473 strain **(E)** is reported in arbitrary unit (au) obtained after quantification of digital images using the Fiji Image J software. Data are expressed as the mean ± STD. ****p < 0.0001 was significant compared to the control group.

The AWME3 ability to disrupt biofilms formed by the tested *K. pneumoniae* strains was further investigated using fluorescence microscopy study of biofilms stained with propidium iodide (PI) (**Figure 3**). The images were obtained using 543/617 nm excitation/emission filter. As shown in control images, tested bacterial strain formed very dense biofilms on glass coverslips. Dense clumps of cells in biofilms were visualized, and bacteria were seen to be heavily colonized and adherent in multiple layers. Exposure to varying concentrations of AWME3 resulted in a significant (p < 0.0001) decrease and scattered bacterial cell presence (**Figures 3B–E**). Upon treating bacterial biofilms with 0.5 MIBC (0.25 mg/ml), the presence of reduced mats, threads, and clumps was observed, with cells displaying dispersion and noticeable gaps between them (**Figure 3B**). AWME3 at a concentration of 0.5 mg/ml (MIBC) significantly decreased the number of cells and the overall bacterial cell count and intensity (**Figure 3C**). The highest impact of AWME3 was clearly observed when the concentration of MIBC was 2 MIBC (1.0 mg/ml) (**Figure 3D**). There was almost 10-fold decrease (p < 0.0001) in fluorescence intensity compared to that of the control group, as shown in **Figure 3E**. These results demonstrate that AWME3 effectively breaks down the pre-existing deposits of extracellular nucleic acids (eNAs) found in mature *K. pneumoniae* biofilms, and the level of disruption is proportional to the used AWME3 concentration.

### 3.6 Disrupted biofilm visualized by scanning electron microscopy

**Figure 4:**
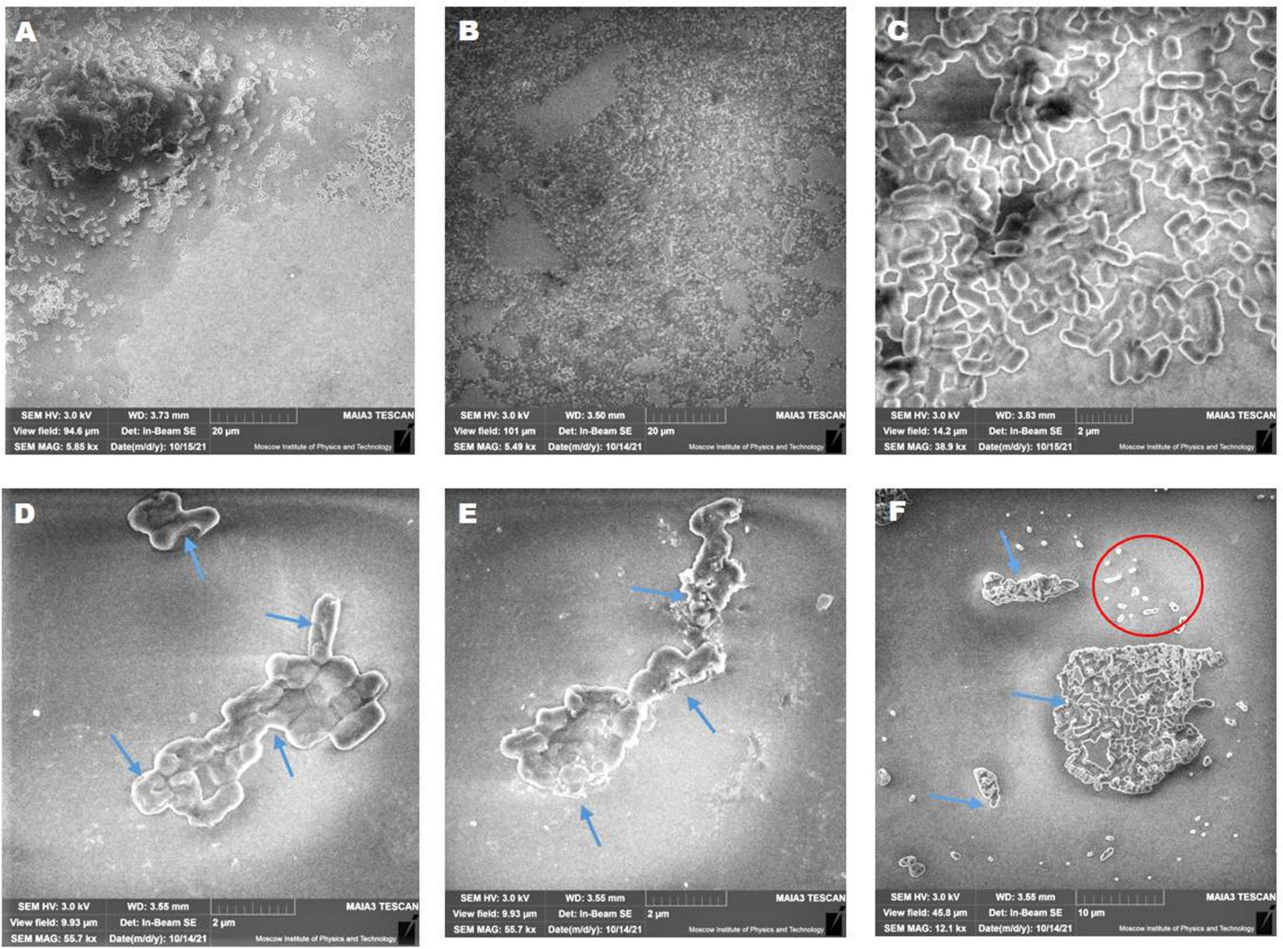
Concentration-related effects of AWME3 on mature biofilm dispersion and biofilm cell death. SEM micrographs of *K. pneumoniae* ATCC BAA-2473 biofilms developed on the glass slide surface incubated for 24 h of incubation at 37°C. Untreated biofilm at different magnifications **(A-C)**; treated biofilms with 0.5 MIBC (0.25 mg/ml) **(D)**; MIBC (0.5 mg/ml) **(E)**; and 2 MIBC (1.0 mg/ml) **(F)** of AWME3. All figures show SEM images with magnification ranging from ×5,490 to ×55,700.

SEM analysis was performed to visually observe the biofilm disruption after AWME3 treatment. Cells appear aggregated and accumulated in multiple layers in untreated biofilms established by *K. pneumoniae* KP ATCC BAA-2473 (**Figures 4A–C**). Furthermore, no morphological alterations were detected in untreated cells, where cells were smooth with intact cell wall and bacilli (**Figure 4C**).

AWME3 treatment caused a significant decrease in the number of adherent bacteria, compared to that of the control cells. The SEM analysis revealed rough surfaces, with wrinkled cell walls and visible pores in the bacterial cells treated with AWME3, as indicated by the blue arrows (**Figures 4D–F**). Lysed cells, cell wall debris, and ghost cells were obvious when biofilms were treated with 2 MIBC (1.0 mg/ml) of AWME3 (blue arrows and red cycle in **Figure 4F**). The biofilm was reduced down to a monolayer of adherent cells, and even single cells were detected (**Figures 4D–F**). These findings suggest that AWME3 is a potent anti-biofilm agent, which can disrupt the mature biofilms.

Thus, three independent microscopy techniques all showed that AWME3 disrupts mature biofilms composed of peptidoglycan layers covered by lipopolysaccharides, lipoproteins, proteins, and eNAs, produced by various MDR hvKp strains on the plastic surface in a dose-related manner.

### 3.7 Hypervirulent *K. pneumoniae* bacteria lose its mucoviscosity in the presence of AWME3

The hypermucoviscous (HMV) phenotype is one of the key virulence factors of *K. pneumoniae*. This phenotype is associated with serious infections such as liver abscesses, pneumonia, and bloodstream infections. The HMV phenotype is characterized by its ability to form a thick, sticky biofilm, which contributes to its pathogenicity (Kawai, 2006). Understanding this phenotype is important for developing effective treatment strategies against *K. pneumoniae* infections.

We conducted an analysis of autoaggregation to test the hypothesis that it plays a role in biofilm formation. Specifically, we investigated how AWME3 affects the autoaggregative behavior of mucoid hypervirulent *K. pneumoniae* strains. The autoaggregation experiment was conducted at room temperature for 24 h. The results are presented in **Supplementary Table S1** and **Supplementary Figure S4A**. The data indicate that 0.5 MIC of AWME3 (0.125 mg/ml) did not have an effect on the autoaggregation of KPi1627, KPM9, and KP ATCC BAA-2473 strains (p = 0.995, p = 0.971, p = 0.945, respectively) (**Supplementary Figure S4A**). Furthermore, the turbidity of the supernatant from the treated cells, measured after cell centrifugation, did not differ from that of the control group (**Supplementary Figure S4B**). Conversely, the same AWME3 concentration resulted in the formation of loose pellets, which differed from the dense pellets formed by all non-treated *K. pneumoniae* strains (**Supplementary Figure S4C**).

For better understanding of the impact of AWME3 on the virulence of hvKp strains, we conducted a straightforward string test considering the positive string as strings longer than 5 mm. The results of the KPi1627 and KPM9 strain tests indicated their virulent nature, except for the KP ATCC strain, which tested negative, suggesting a lack of virulence. Of note, the untreated KPi1627 strain showed the highest HMV-phenotype among all the tested bacteria, with a string 51.7 ± 3.5 mm in length. In comparison, the KPM9 and KP ATCC BAA-2473 strains had string lengths of 31 ± 3.63 and 3.81 ± 1 mm, respectively (**Supplementary Table S2**, **Supplementary Figure S5**). It is noteworthy that when all string-positive isolates were exposed to 0.5 MIC (0.125 mg/ml) of AWME3, they became negative in the string test highlighting the high efficacy of AWME3 in combating one of the key virulence factors of *K. pneumonia* strains with HMV phenotype.

### 3.8 Effect of AWME3 on rudimentary motility of *K. pneumoniae* strains

Throughout history, *K. pneumoniae* has been recognized as a major cause of urinary tract infections (UTIs) further emphasizing its significance beyond its association with pneumonia (Paczosa and Mecsas, 2016). The expression of fimbriae is crucial for the successful colonization of the urinary epithelium by *K. pneumoniae*. These structures facilitate attachment to urothelial cells and play a pivotal role in promoting bacterial adhesion to abiotic surfaces, like urinary catheters (Stahlhut et al., 2012). Bacterial motility is a critical factor in the successful colonization of both living and non-living surfaces. Remarkably, the hyper fimbriae phenotype appears to grant the mutant strain a form of rudimentary mobility. While *K. pneumoniae* was previously considered to be non-motile, the discovery of rudimentary (limited) motility in this bacterium is well documented (Carabarin-Lima et al., 2016; Érika et al., 2021) representing another phenotype associated with virulence in *K. pneumoniae* infections.

Therefore, we conducted an assessment of the sub-MIC (0.125 mg/ml) impact of AWME3 on the three types of rudimentary motility (swimming, swarming, and twitching) of *K. pneumoniae* isolates. This experiment analysis showed that AWME3 extract has a significant effect on the basic (twitching) mobility of *K. pneumoniae* strains KPi1627, KPM9, and KP ATCC BAA-2473 (**Supplementary Table S** **3**). In particular, the AWME3 extract significantly reduced the swimming motility of KPM9 and KP ATCC BAA-2473 strains (p = 0.0025, p < 0.0001, respectively).

**Figure 5:**
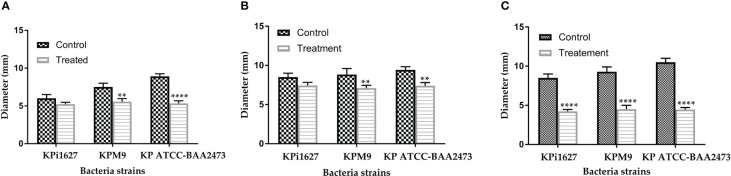
Effect of sub-MIC of AWME3 on rudimentary motility of *K pneumoniae* strains. **(A)** Mean swimming motility, **(B)** mean swarming motility, and **(C)** mean twitching motility of KPi1627, KPM9, and KP ATCC BAA-2473 isolates. Data are mean values ± STD (n = 3). Data were analyzed by two-way ANOVA, followed by Sidak’s multiple comparisons test; p-value ranged between **p = 0.003 and ****p < 0.0001.

However, no notable effect on KPi1627 swimming motility was observed at 0.5 MIC (0.125 mg/ml) of AWME3. The swarming motility of MDR KPM9 and KP ATCC BAA-2473 strains was significantly (p = 0.007, p = 0.003) reduced after being exposed to 0.5 MIC (0.125 mg/mL) of AWME3. The sub-MIC (0.125 mg/ml) of AWME3 significantly (p < 0.0001) reduced the twitching motility zone diameters of all *K. pneumoniae* strains (**Supplementary Figure S6**, **Figure 5C**). Furthermore, AWME3 at sub-MIC levels decreased the twitching motility of KPi1627, KPM9, and KP ATCC BAA-2473 bacterial strains by approximately 50% of the zone diameters. The treated bacterial strains exhibited twitching motility in the range of 4.23 ± 0.25 to 4.47 ± 0.25 mm, while the non-treated control groups showed significantly higher motility ranging from 8.5 ± 0.5 to 10.5 ± 0.5 mm (**Supplementary Table S** **3**, **Supplementary Figure S** **6**; **Figure 5C**).

### 3.9 Suggested mechanism of AWME3 actions against *K. pneumoniae* strains grown under planktonic bacterial mode

#### 3.9.1 AWME3 impact on permeability of bacterial cell membranes

To verify if the changes in bacterial membranes caused by AWME3 affected their permeability, we conducted experiments using crystal violet (CV) and ethidium bromide (EtBr) uptake tests. Unlike CV, EtBr is able to accumulate in bacterial cells by either increasing membrane permeability or by inhibiting efflux pumps. Before testing for CV uptake, the bacteria were exposed to AWME3 in different concentrations (ranging from 0.0625 to 0.5 mg/ml) for 4 h. Subsequently, exposure was extended to concentrations from 0.25 to 0.5 mg/ml for 8 h to test the uptake of EtBr. We measured the amount of EtBr that entered the cells instead of looking at how much was removed through the membrane. After 15 min, we quantified and analyzed the EtBr fluorescent signal.

**Figure 6:**
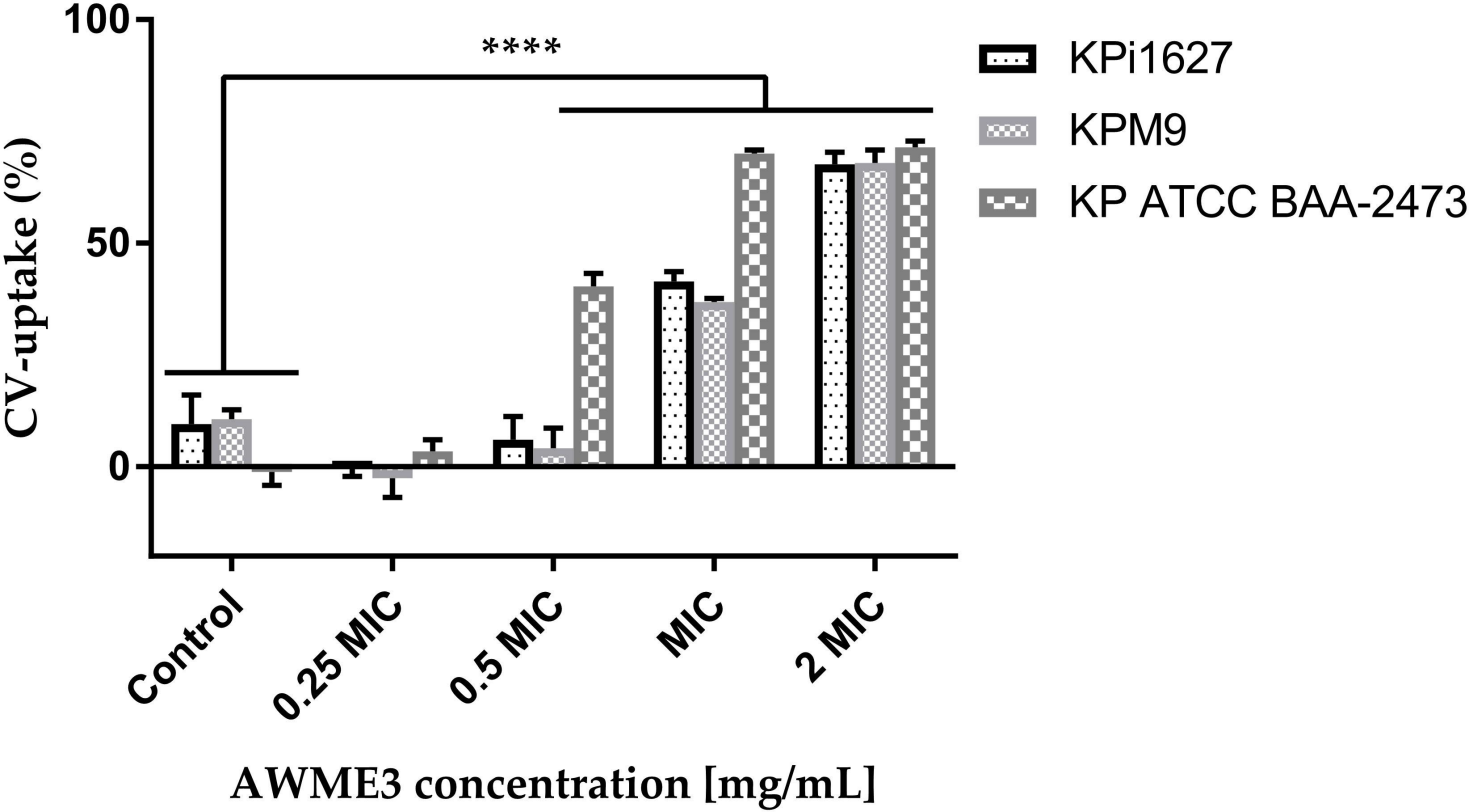
Effect of AWME3 on CV uptake through cell membranes of different strains of *K. pneumoniae*. All tested bacterial strains treated with various concentrations 0.25 MIC (0.0625 mg/ml), 0.5 MIC (0.125 mg/ml), MIC (0.250 mg/ml), and 2 MIC (0.5 mg/ml) of AWME3 for 4 h. Action on membrane permeability calculated after measuring the absorbance of crystal violet dye. All data are expressed as mean ± SD of three independent experiments. Statistical analysis performed using two-way ANOVA variance and Dunnett’s multiple comparisons test (****P=0.0001).

CV easily penetrates and traverses the only damaged cell membrane (Li et al., 2013). We discovered that the *K. pneumoniae* strains treated with AWME3 exhibited varying levels of CV-uptake activity (**Figure 6**). Out of all the strains tested, KP ATCC BAA-2473 displayed remarkable susceptibility to AWME3. The study revealed a substantial (p < 0.0001) increase in CV uptake to 40.4%, 70.05%, and 71.43% at 0.5 MIC, MIC, and 2 MIC of AWME3, respectively, in comparison to untreated bacteria (1.15%) (**Figure 6**). When the highly virulent MDR KPi1627 and KPM9 strains were treated with a low dose of 0.5 MIC AWME3 (0.125 mg/ml), their membrane permeability did not change much. The KPi1627 recorded 6.05% and KPM9 recorded 4.13%, compared to their control groups at 9.6% and 10.7%, respectively. However, at an MIC concentration 0.25 mg/ml, the permeability of the cell membranes of KPi1627 and KPM9 cells was greatly increased (p < 0.0001) reaching 41.48% and 36.83% respectively. The highest concentration of 2 MIC (0.5 mg/ml) caused also significant (p < 0.0001) membrane permeabilization of 67.7% and 67.9% for the same cells, as shown in **Figure 6**.

Thus, we proved AWME3 permeabilization or disruption of the bacterial cells membrane allowing EtBr to enter the cell cytoplasm. Treatment with various concentrations of AWME3 for 8 h resulted in lower EtBr emission intensity compared to that of the control indicating significant EtBr uptake (**Supplementary Figure S7A**). This result supports our findings on CV uptake. EtBr uptake values for KPi1627, KPM9, and KP ATCC BAA-2473 strains changed subtly and more significantly when exposed to 0.5 MIC (0.125 mg/mL) and MIC (0.25 mg/mL) of AWME3, respectively (**Supplementary Figure S7** **B**). The increases were 60.56%, 55.98%, and 56.37%, respectively, at the MIC concentration. Notably, the values for hvKp strains were significantly higher reaching 80.43%, 77.2%, and 76.88% when exposed to 2 MIC (0.5 mg/mL) of AWME3, as shown in **Supplementary Figure S7B**. KP ATCC BAA-2473 strain exhibited the highest susceptibility to AWME3 compared to the other strains. The aforementioned results clearly demonstrate that AWME3 can effectively increase permeability of the cell membranes of all *K. pneumoniae* strains in a dose-related manner. These findings have been confirmed through CV and EtBr uptake assays.

#### 3.9.2 AWME3 impact on cell wall Lewis acid–base or electron-acceptor/electron-donor characteristics of *K. pneumoniae* strains

Biofilm formation studies have shown that electrostatic and van der Waals interactions play an important role on cell adhesion along with the growing importance of Lewis acid–base interactions (Vernhet and Bellon-Fontaine, 1995). The microbial adhesion to solvents (MATS) is one of the simple and reliable method to gather information about van der Waals and Lewis acid–base or electron-acceptor/electron-donor characteristics influenced on bacterial cell adhesion. It was inspired by the MATH (microbial adhesion to hydrocarbons) method first described 40 years ago and being the golden standard for bacterial cell surface hydrophobicity until to date (Rosenberg, 1984).

In this study, we demonstrated that tested bacterial species showed different degrees of microbial adhesion to solvents (hydrophobicity). **Supplementary Table S4** highlights the higher rates of cell adhesion to chloroform, which is an acidic solvent, compared to both ethyl acetate, a strongly basic solvent, and toluene. No clump or lysis of cells has been observed by phase-contrast microscopy. All untreated strains showed the highest affinity for the acidic solvent and a low affinity for the basic solvents. The adhesion levels to n-alkane (hexane) were uniformly low across all bacteria, with the KPM9 strain showing an adhesion of 6.62 ± 5.95%, while the KPi1627 and KP ATCC BAA-2473 strains displayed noticeably lower adhesion levels of 2.42 ± 3.25% and 1.09 ± 2.7% respectively (**Supplementary Table S4**). These results showed that, without treatment, the cell surfaces of all strains had a slightly higher electron-donating (basic) nature than electron accepting (acidic).

**Figure 7:**
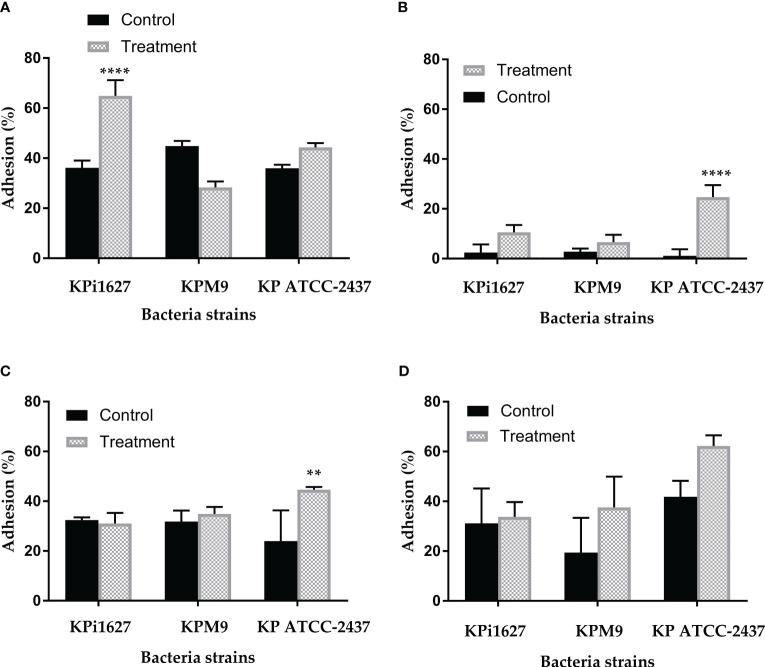
The influence of sub-MIC of AWME3 on tested bacterial strain hydrophobicity. The effect of 0.5 MIC (0.125 mg/ml) of hydrophobicity activity of KPi1627, KPM9, and KP ATCC BAA-2473 against different hydrocarbons, **(A)** chloroform, **(B)** hexane, **(C)** ethyl acetate, and **(D)** toluene. Data are mean values ± STD (*n* = 3). Data were analyzed by two-way ANOVA, followed by Tukey’s multiple comparisons test; p-value ranged between **p = 0.009 and ****p < 0.0001.

In contrast, the treatment with 0.5 MIC (0.125 mg/ml) of AWME3 resulted in a significant (p < 0.001) increase in adhesion to chloroform (an acidic solvent): from 36.31 ± 2.84% and 35.99 ± 1.37% up to 64.9 ± 6.26% and 44.28 ± 1.7% for KPi1627 and KP ATCC BAA-2473 strains, respectively (**Supplementary Table S4**; **Figure 7A**). Moreover, both of these strains exhibit a significant increase in their ability to adhere to a polar n-alkane (hexane) solvent, with adhesion percentages reaching up to 10.6 ± 2.9% and 24.72 ± 4.75% for KPi1627 and KP ATCC BAA-2473 strains, respectively (**Supplementary Table** **S4**). The KPM9 strain kept its affinity to acidic, basic, and a polar n-alkane (hexane) solvent unchanged after treatment. However, there was a small increase in its affinity to toluene from 19.42 ± 4.4% to 37.55 ± 2.86% (**Supplementary Table S4**; **Figure 7D**). Thus, AWM3 treatment increased electron-donating properties of cell surfaces in all strains, except for KP ATCC BAA-2473, which had slightly higher electron-accepting characteristics after treatment (**Supplementary Table S4**; **Figure 7C**). The substantial 4- and 20-fold rise in microbial adhesion to n-alkanes for KPi1627 and KP ATCC BAA-2473 strains, respectively, clearly demonstrates the significant enhancement of the van der Waals property of bacterial cell membranes after AWME3 treatment.

## 4 Discussion

*K. pneumoniae* is a critical pathogen responsible for a variety of infections in the hospital environment particularly in intensive care units where it causes nosocomial infections. Our previous research has revealed that the clinical isolate *K. pneumoniae* KPi1627 and the environmental isolate *K. pneumonia* KPM9 exhibited resistance to colistin but remained susceptible to the quinolone group. Conversely, the standard NDM-1 *K. pneumoniae* ATCC BAA-2473 strain was resistant to the quinolone group but sensitive to colistin. Moreover, all three strains demonstrated sensitivity to doxycycline. The AWME3 extract, obtained from HI larvae fat, not only inhibits but also eliminates all tested *K. pneumoniae* strains when grown in planktonic cell mode. This effective action occurs at a minimum inhibitory concentration (MIC) and a minimum bactericidal concentration (MBC) of 250 µg/ml (Mohamed et al., 2022).

The endurance of biofilm has proven to be a challenging task due to the heightened resistance exhibited by biofilms when subjected to microbiocides and antibiotics in contrast to planktonic cells (Panebianco et al., 2021). Most clinical isolates of *K. pneumoniae* contain two types of fimbrial adhesions, such as types 1 and 3 fimbriae, which are important for *K. pneumoniae* pathogenicity and biofilm formation (Schroll et al., 2010). In addition, all tested bacteria were MDR pathogens (Lev et al., 2018; Chmielewska et al., 2020) comparing bacterial strains with their antibiotic resistance. All strains were found to form strong biofilms and remain resistant. These findings aligned with several other studies (Nirwati et al., 2019; Ochońska et al., 2021; Oleksy-Wawrzyniak et al., 2022). Although demonstrating strong bacteriostatic (MIBC > 50 µg/ml) efficacy against all tested bacterial strains, the standard antibiotic (Dox) at even 4 mg/ml was unable to break down the mature biofilms. On the contrary, the AWME3 extract at a concentration of 1 mg/ml exhibits significant antimicrobial properties against mixed, single, and mature biofilms (see **Supplementary Figure S6**; **Figures 1**, **2**) formed by various multidrug-resistant strains of *K. pneumoniae*, including KPi1627, KPM9, and KP ATCC BAA-2473.

During biofilm formation, eDNA mediates bacterial attachment to surfaces (Whitchurch et al., 2002), and it also plays a major role in mature biofilms. The importance of eDNA in biofilm formation has been proven by the fact that DNase I inhibits biofilm formation or detaches existing biofilm of several Gram-positive and -negative bacterial species (Okshevsky and Meyer, 2013). We used three different microscopy techniques in our study, and all of them consistently showed that AWME3 disrupts mature biofilms, as depicted in **Figure 2**.

For the first time, we have demonstrated the effectiveness of AWME3 in combat with two key virulence factors of *K. pneumoniae* strains. Most of the hvKp strains typically possess a thick, hypermucoid capsule and therefore produce mucoid colonies that generate a positive result in string test; therefore, hypermucoviscous phenotype is mostly associated with hypervirulence (Shon et al., 2013). Lev et al. (2018) reported that KPM9 and KPi1627 have capsular type K20 and K2, respectively. The capsule is a crucial virulence factor that enhances hvKp’s resistance to various antibacterial agents and its ability to form a biofilm. This biofilm, in turn, grants the bacterium resistance to antibiotics and protects it during periods of starvation stress (Wu et al., 2011; Zheng et al., 2018). Failing to eradicate or reduce the virulence level of hvKp capsules by the previous generation of antibiotics led to the weakening of the last-resort antibiotics, thus emphasizing the urgent necessity for identifying safe and selective therapeutic agents aimed at preventing and treating resistant bacterial strains (Ling et al., 2015). In the present study, we have successfully demonstrated the remarkable effectiveness of AWM3, when used at a sub-MIC concentration of 0.125 mg/ml, in effectively eliminating hypermucoviscosity (**Supplementary Table S2**, **Supplementary Figure S5**). Hypermucoviscosity is a crucial virulence factor of *K. pneumoniae*, and our findings highlight the immense potential of AWM3 in addressing this problem.

For a considerable period, *K. pneumoniae* was perceived as non-motile Gram-negative rod bacilli. However, this perspective was challenged when Lima and León-Izurieta (Carabarin-Lima et al., 2016) demonstrated the presence of polar flagella in *K. pneumoniae* isolated from a patient with neonatal sepsis. Furthermore, they described a swimming-like motility phenotype generated by flagella in these clinical isolates. The hyperfimbriated phenotype represents a rudimentary form of motility serving as another virulence factor of *K. pneumoniae*. Moreover, the *K. pneumoniae* genome contains the *flk* gene, which encodes a regulator of flagella biosynthesis. The observed rudimentary movement in the mutant strain is the result of type 1-like fimbriae production or the KpfR regulator enhancing the expression of flagellar genes. Sharma et al. (2019) conducted a comprehensive study on comparative proteomics and systems biology. Their research focused on investigating the correlation between the decrease in proteins related to motility (such as flagella, fimbriae, and pili) and the formation of biofilms. This correlation is significant as it has the potential to contribute to the development of drug resistance. Our findings reveal that even at a sub-MIC concentration (0.125 mg/ml), AWM3 effectively suppresses (**Figure 5**) the twitching motility generated by all bacteria strains (Carabarin-Lima et al., 2016; Érika et al., 2021). In this regard, our data are consistent with previous studies that have shown the significant reduction in swimming and swarming motility of various strains of human pathogens when treated with natural product extracts (Song et al., 2019; Balkrishna et al., 2021; Vishwakarma et al., 2022).

Long-chain free fatty acids (FFAs) have the potential to neutralize the virulence factors of bacterial pathogens (Borreby et al., 2023). Certainly, various FAs imitate virulence factors and regulate the motility, fimbriae, hyphae, and biofilm formation of various microorganisms. For instance, oleic acid, which was found as a component of AWM3, inhibited swarming motility and pyocyanin production in *Pseudomonas aeruginosa* (Singh et al., 2013). Several publications stated that single or combined fatty acids disrupt and eradicate biofilms formed by MDR pathogenic bacteria strains (Singh et al., 2013; Eder et al., 2017; Hobby et al., 2019). Our extract AWME3 shows higher activity compared to the extract of *Withania somnifera* seeds, which contains a large amount of fatty acids (Balkrishna et al., 2021). AWME3 was also more potent than different essential oils used to disrupt New Delhi metallo-β-lactamase-1-producing uropathogenic *K. pneumoniae* strains (Kwiatkowski et al., 2022). Besides, AWM3 was superior to mechanically processed oils from *Hermetia illucens* larvae and *Bombyx mori* pupae in their ability to kill bacterial cells (Saviane et al., 2021). Previous studies stated that natural products, in particular saturated fatty acids (SFAs) and polyunsaturated fatty acids (PUSFAs), were able to disrupt and inhibit mature biofilms established by *K. pneumoniae* strains (Jiang et al., 2020; Kumar et al., 2020; Galdiero et al., 2021; Saifi et al., 2024).

The SEM analysis revealed wrinkled cell walls and visible pores in the bacterial cells treated with AWME3 (**Figure 4**) confirming our previous report (Mohamed et al., 2022) that AWME3 likely targets the bacterial cell wall membranes. The results of the CV (**Figure 6**) and EtBr-uptake (**Supplementary Figure S7A**) assays clearly demonstrate that these membrane disturbances can be accompanied by an increase in the permeability of the cell membranes of all *K. pneumoniae* strains in a dose-related manner. Alterations in cell morphology and viability are consistent with other studies that have shown the ability of fatty acids and their glycerides to inhibit and eliminate single or mixed biofilms formed by various types of microorganisms through leakage in the cell wall/cell membrane. Furthermore, these substances impair the electron transport chain, block enzymes, and cause deficiencies in nutrient uptake [ (Baker et al., 2018; Hobby et al., 2019; Kumar et al., 2020; Galdiero et al., 2021). The impact of exogenous fatty acids (linoleic acid, γ-linolenic acid, α-linolenic acid, arachidonic acid, eicosapentaenoic acid, dihomo-γ-linolenic acid, and docosahexaenoic acid) on *K. pneumonia* was explored in the study conducted by Hobby et al. (2019). The study also involved the treatment of *K. pneumoniae* with antimicrobial peptides (AMPs). Contrary to our current study, the research discovered that supplementing the medium with fatty acids resulted in a significant rise in the growth of *K. pneumoniae*. However, these exogenous FAs also caused structural changes in the phospholipids. This raises the intriguing possibility that these modifications could potentially enhance membrane permeability, which aligns perfectly with our current data.

To understand the mechanistic pathways of AWME3-mediated anti-virulence and anti-biofilm activity, we investigated the Lewis acid–base or electron-acceptor/electron-donor characteristics, as well as the van der Waals interactions within the bacterial cell wall. By examining the surface properties of microorganisms, we aim to better understand how to effectively reduce or prevent their adhesion. For this purpose, the standard microbial adhesion to solvents (MATS) technique was used as the only simple and reliable method to gather information about the acid–base properties of microbial cells. Our data shows that, if left untreated, the cell surfaces of all strains displayed a slightly higher electron-donating (basic) nature compared to electron-accepting (acidic) properties. However, when treated with 0.5 MIC (0.125 mg/ml) of AWME3, the electron-donating properties of cell surfaces in all strains were significantly enhanced, except KP ATCC BAA-2473. Interestingly, KP ATCC BAA-2473 exhibited a slightly higher electron-accepting characteristic after treatment (**Supplementary Table S4**, **Figure 7**). The same treatment resulted in significant enhancement of the van der Waals interactions of bacterial cell membranes as indicated by a substantial increase in microbial adhesion to n-alkane for KPi1627 and KP ATCC BAA-2473 strains. The hydrophobicity of the microbial cell surface is an important factor in the adhesion phenomenon (Rosenberg et al., 1991; AChinas et al., 2019). There is a strong correlation between biofilm and cell surface hydrophobicity. The hydrophobic/hydrophilic nature of the surface is determined by the percentage of cells that are attached to n-alkanes. The surface is considered relatively hydrophobic when this percentage exceeds 50% and relatively hydrophilic when it is lower than 50% (Bellon-Fontaine et al., 1996). Hence, it is likely that the treatment with AWME3 reduces the hydrophilic nature of the bacterial wall membranes of *K. pneumoniae*.

From a mechanistic standpoint, AWME3 appears to behave differently than group 2 capsule polysaccharide (G2cps), which is another promising candidate for combating virulence and biofilm formation (Bernal-Bayard et al., 2023). *Klebsiella’s* CPS has dual effects during biofilm formation helping with initial adhesion, maturation, but repelling competitors. It was proposed that CPS alters the physical properties of abiotic surfaces by increasing its hydrophobicity (Dos Santos Goncalves et al., 2014). The anti-biofilm activity of G2cps is due to changes in ionic charge and Lewis base properties induced by the CPS polysaccharides in membranes of *Escherichia coli* cells (Travier et al., 2013). In contrast to AWME3, which significantly increases the Lewis base properties of *K. pneumoniae* membranes (**Figure 7**), G2cps reduced the *E. coli* affinity to chloroform by 35%, thus indicating that contact with G2cps strongly reduces bacterial Lewis base properties. It is worth noting that *E. coli* mutants with partial resistance to G2cps, when exposed to G2cps, displayed higher Lewis base properties compared to G2cps-susceptible WT-*E.coli* cells. This suggests that treating *K. pneumoniae* cells with AWME3 may cause changes in their response to CPS by increasing the Lewis base properties of the bacterial cell wall membranes. It is noteworthy that among all other FAs, AWME3 contains cis-2-decanoic acid and cis-9-octadecenoic acid, which have been reported to be more effective against biofilms formed by methicillin-resistant *Staphylococcus aureus* (MRSA) (Mirani et al., 2017). After being exposed to these fatty acids, the established biofilms were dispersed, and the surviving cells could not regain their biofilm lifestyle. Wild-type MRSA strains can produce fatty acid-modifying enzyme (FAME) to inactivate the bactericidal activity of fatty acids by esterification to cholesterol. The biofilm indwellers are non-metabolically active and incapable of synthesizing FAME rendering them susceptible to the anti-biofilm properties of cis-2-decanoic acid and cis-9-octadecanoic acid (Mirani et al., 2017). Hence, bacteria that create biofilms and produce little FAME are more likely to be vulnerable to natural anti-virulence agents like AWME3.

## 5 Conclusion

In conclusion, the unique combination of natural FAs in our AWME3 extract, rather than an individual FA, appears to be responsible for effectively combating biofilms and two key virulence traits in the tested MDR hvKp strains of *K. pneumoniae*. Unlike other proposed anti-virulence methods and agents, AWME3 not only possesses bactericidal properties but also effectively reduces the hydrophilic quality of the bacterial wall membranes of *K. pneumoniae*. This remarkable compound serves as a trustworthy anti-biofilm agent against both mucoid and non-mucoid hvKp strains, and potentially other multidrug-resistant (MDR) bacterial pathogens. This discovery will help to identify new candidates, like AWME3, that can be used as anti-virulence agents with a reduced risk of developing resistance. These agents have the potential to effectively treat multidrug-resistant nosocomial bacterial infections and oral bacteria that can form biofilms.

## Data availability statement

The original contributions presented in the study are included in the article/**Supplementary Material**. Further inquiries can be directed to the corresponding authors.

## Author contributions

HM: Conceptualization, Data curation, Formal analysis, Investigation, Methodology, Software, Visualization, Writing – original draft, Validation. EM: Supervision, Validation, Writing – review & editing, Visualization, Resources. MD: Funding acquisition, Writing – review & editing. SL: Project administration, Validation, Writing – review & editing, Resources, Visualization.
