# Supplementary material for: A unique combination of natural fatty acids from Hermetia illucens fly larvae fat effectively combats virulence factors and biofilms of MDR hypervirulent mucoviscus Klebsiella pneumoniae strains by increasing Lewis acid–base/van der Waals interactions in bacterial wall membranes
Source: Front Cell Infect Microbiol. 2024 Jul 25;14:1408179. doi: 10.3389/fcimb.2024.1408179 (PMC11306206; doi:10.3389/fcimb.2024.1408179)
Supplement: Supplementary file 1 [file DataSheet_1.pdf]

frontiers

Supplementary Material

Supplementary methods

Ethidium bromide (EtBr) uptake

This assay was performed according to Hobby *et al.* (Hobby et al., 2019) with minor changes, where *K. pneumonia* strains were harvested and washed with PBS, pH 7.4 three times. The pellets were resuspended in PBS, adjusted to OD600 ~ 0.5, and 200 µL of suspension was added in triplicates to 96-well black clear bottom plates where it was mixed with AWME3 at the final concentration from ¼ MIC to 2 MIC for 8 h. Next, bacterial cells were incubated with EtBr (5 µg/mL), in the dark for 15 min. After centrifugation, the supernatant was analyzed using an excitation/emission maxima of 330/585 nm. Data normalization was performed on a control sample containing EtBr without bacteria and representing the maximal fluorescence intensity using a CLARIOstar® *Plus* multimodal plate reader. The experiment was conducted in triplicate and by three biological replicates.

Autoaggregation assay

Autoaggregation considered as cell clumping and sedimentation was measured as previously described (Chiarelli et al., 2020) with minor changes. All *K. pneumoniae* strains were grown overnight in LB broth at 37 °C with shaking at 200 rpm/min. After centrifugation at 3000×g for 7 min, bacterial pellets were re-suspended in PBS, pH 7.4 to get an OD_{600} ~ 0.5 ($OD_{initial}$). Next, cells were treated with sub-MIC (0.125 mg/mL) of AWME3, treated and untreated (control) bacteria were incubated for 6 h at 37 °C in a static condition, then the OD_{600} of the upper layer of each bacterial suspension was measured at OD_{600} (OD_{final}). Autoaggregation (%) was calculated using the following equation: Auto-aggregation (%) = $(1 – OD_{final}/OD_{initial}) \times 100$.

Mucoviscosity, string, and precipitation assays

The mucoviscosity of *Klebsiella pneumoniae* strains was determined by performing the sedimentation assay as previously described (Mondal et al., 2016; Xu et al., 2021). Briefly, overnight culture grown in LB broth was diluted to 0.1 OD_{600} in media and grown at 37 °C with or without the sub-MIC (0.125 mg/mL) of AWME3. After that, the culture was incubated overnight, and then centrifuged for 5 min at 1000×g. The supernatant was removed without disturbing the pellet for OD_{600} measurement, left in standing condition for 15 min, and later visually tested for mucoviscosity supernatant and dense pellet. The hypermucoviscous phenotype of *K. pneumoniae* isolates were confirmed by string test. The bacterial strains were grown on an agar plate at 37 °C for 72 h, and the formation of a mucoviscous string measuring >5 mm on a bacteriology inoculation loop was defined as a positive string test (Lin et al., 2020).

Microbial adhesion to solvents (MATS) assay

The microbial adhesion to organic solvents test was performed to evaluate the Lewis acid-base properties and the hydrophilic/hydrophobic nature of bacterial surfaces after AWME3 treatment. The cell surface hydrophobicity of biofilm-forming *K. pneumoniae* strains was determined in the presence

of AWME3 according to Bellon-Fontaine *et al*. (Bellon-Fontaine et al., 1996). This partitioning method relies on comparing microbial cell affinity to a monopolar solvent against to the polar solvent. The monopolar solvent can either be acidic (electron-accepting) or basic (electron-donating). However, it is crucial for both solvents to possess similar Lifshitz-van der Waals surface tension components. Hence, we selected the following pairs of solvents to determine the acidic and basic properties of microbial cells using the MATS method: i) chloroform, an acidic solvent that exhibits negligible basic character when pure and hexane; ii) ethyl acetate, a strongly basic solvent and toluene. Hexane is an apolar n-alkane. The adsorption of toluene molecules to the cell surface depended on cell surface hydrophobicity in the first 0.5h. The solvents used in this study were sourced from Sigma and were of the highest purity grade.

Briefly, a total of 10^9 cells were subjected to a sub-MIC treatment of AWME3 (125 µg/mL) for a duration of 4 hours at a temperature of 37 °C. As a control, an equivalent number of cells were left untreated. Cells were centrifuged at 4000 rpm for 5 min and the resulting pellets were washed twice and resuspended in 1.2 mL suspending liquid (potassium phosphate buffer at concentrations of 0.1 M and adjusted to pH 7). The optical density was measured at 400 nm of the bacterial suspension before mixing. The bacterial suspension was vortexed for 90 seconds with 0.2 mL of the solvent (hexane, ethyl acetate, chloroform, and toluene) being studied. After 15 minutes of standing, the mixture was carefully examined to ensure that the two phases were completely separated. A precise sample of 1 mL was then taken from the aqueous phase and its optical density was measured at 400 nm. We monitored the aqueous phase using phase contrast microscopy to detect any cell clumping or lysis that could have been caused by the solvent. The percentage of adhered cells was then determined using the formula % adherence = (1 – A/Ao) x 100, where Ao represents the initial optical density measured at 400 nm of the bacterial suspension before mixing and A represents the absorbance after mixing.

In vitro biofilm formation (BF) determination

Biofilm formation was determined in 96-well polystyrene microtiter plates using crystal violet method described by Ramos- Vivas *et al*. (Ramos-Vivas et al., 2019). *K. pneumoniae* strain cultures were grown in LB broth overnight at 37 °C, and then adjusted to 0.1 OD_{600} nm without shaking for 24 h at 37 °C. The planktonic cells were removed, and washed carefully with PBS pH 7.2±0.2 without disturbing biofilm formed along the wall of the plate, or attached on the bottom, then dried at 37 °C for 40 min. After that, 125 µl of 1.5% CV was added to each well and incubated at room temperature for 15 min. The plate was washed three times with d.H_2O to remove the stain, then, drayed at 60 °C for 1 h. Next, 125 µl of 30% acetic acid was added to each well, and then incubated at room temperature for 10 min with shaking at 400 rpm/min. The absorbance was measured at 550 nm. BF was determined according to the formula: BF= AB - CW, where AB is the OD of the wells attached by bacteria, and CW is the OD of the stained control wells (no bacterial cells). The experiment was performed in triplicate on three different days. Biofilm production capacity by each strain was scored as either strong (BF≥0.300), moderate (BF=0.200-0.299), weak (BF=0.100-0.199), or negative (BF≤0.100 (Al-Marri et al., 2021).

Action of AWME3 on the biofilm formation

Biofilm formation inhibition assay was performed by a modified protocol described by Seukep *et al*. (Seukep et al., 2020) for quantitation of crystal violet in a 96-well plate. All *K. pneumoniae* strains were cultured overnight at 37 °C, then harvested, washed three times with PBS, pH 7.4, and adjusted to 10^6 CFU/mL of suspension. The 2-fold dilution in LB broth of 100 µL AWME3

was performed in a 96-well plate to give the final four concentrations 1, 0.5, 0.25, 0.125 mg/mL of AWME3. For negative control, 200 µL of LB broth was added to the 96-well plate in triplicate. Next, 100 µL of bacterial suspension was added to each well except the negative control, then incubated at 37 °C for 24 h without shaking. Planktonic cells were removed by washing with PBS, pH 7.2 three times, followed by drying at 37 °C for 40 min. After that, 125 µL of 1.5% CV was added to each well and incubated at room temperature for 15 min. The plate was washed 3 times with d.H_2O to remove the excessed stain, following by draying at 60 °C for 1 h. Next, 125 µL of 30% acetic acid was added to each well, then incubated at room temperature for 10 min with shaking at 400 rpm/min. The absorbance was measured at 550 nm. All tests were performed in triplicates, and repeated three times. The percentage of biofilm inhibition was calculated using the following formula:
% of biofilm inhibition = [$OD_{control} - OD_{treated} / OD_{control}$] × 100

Mixed biofilms disrupted by AWME3

The disruption of the mixed biofilm assay performed according to Lara *et al*. (Lara and Lopez-Ribot, 2020) with some modifications. AWME3 was prepared by 2-fold dilution in 96-well microtiter plate to give final concentrations in the range of 0.08-1.0 mg/mL. All three *K. pneumoniae* were grown in LB broth media, and then harvested, the pellet was washed twice with PBS, and each strain was adjusted to 10^6 CFU/ mL density. The mixed bacterial culture was prepared by adding an equal volume (1.0 mL) of each strain (1:1:1) in the same tube. The 0.1 mL of mixed bacteria with suspension 10^6 CFU/mL was introduced to each well of the 96-microplate. The negative control was LB broth only and the positive control was the mixed biofilms without treatment. The microtiter plate was sealed to prevent evaporation, followed by incubation at 37 °C for 24 h. The planktonic cells in the mixed biofilm were removed during gentle washing with PBS, dried, stained with 125 µl of 0.1% CV solution, and incubated at room temperature for 15 min. The excess stain was removed, then wells were rinsed with dH_2O, dried, and finally, 125 µl of 30% acetic acid was added to each well to dissolve the mixed biofilm to be measured at 570 nm. The % of the inhibition of the mixed biofilm was calculated by the following formula: % mixed biofilm reduction [$OD_{control} - OD_{assay} / OD_{control}$] × 100.

Biofilm visualization via light microscopy

The ability of AWME3 extract to inhibit biofilm formed by *K. pneumoniae* ATCC BAA-2473 strain was confirmed by a microscopic technique (Sateriale et al., 2020). Briefly, the biofilm was grown on glass cover slides (1 cm^2) placed in 6-well polystyrene plates. In particular, aliquots of 1.0 mL of bacterial culture was adjusted 0.2 OD_{600}, then it was dispensed on the cover glass, incubated for 6 h in static conditions at 37 °C. After that, the wells were filled with 0, 125, 250, and 500 µg/mL of AWME3 concentrations, reaching a final volume of 2 mL in each well, before aerobic incubation at 37 °C for 24 h without shaking. The bacterial cultures without treatment were used as a negative control. After incubation, wells were emptied, washed with PBS three times to remove the planktonic cells, fixed with 90% ethanol for 15 min, and completely dried at 30 °C. Then, biofilms were stained with 1% CV for 20 min at room temperature. The excess dye was washed with d. H_2O. Finally, dried stained glass covers were placed on slides and were observed at 40X magnification of the light microscopy (Nikon, Model: ECLIPSE TS 100, Tokyo Boeki, Japan).

Supplementary Figures

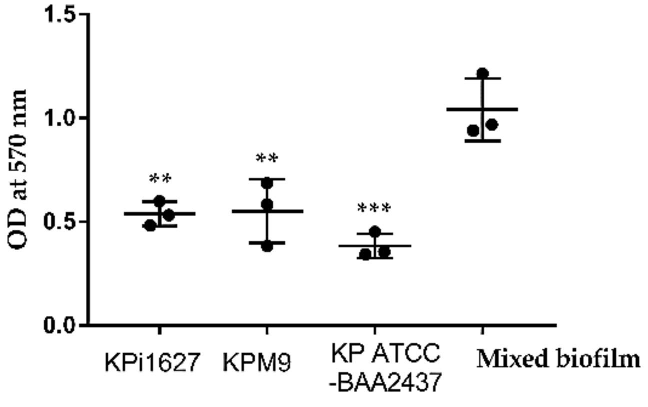

Supplementary Figure S1. Quantitation of hvKp strains formed biofilm on polystyrene microtiter plates (static conditions) after 24 h. Biofilm formation (BF) was measured by crystal violet at 570 nm (optical density, OD_{570}). Biofilm establishment by each isolate was scored as either strong (BF≥0.300), moderate (BF=0.200-0.299), weak (BF=0.100-0.199), or negative (BF<0.100). Data were analysed by the one-way ANOVA test with Tukey's multiple comparison test. Results indicated as means ± STDs. Asterisks indicate statistical significance (**p =0.003, ****p < 0.0005), when different bacteria strains KPi1627, KPM9, and KP ATCC-BAA2437 were compared with mixed biofilm of these strains.

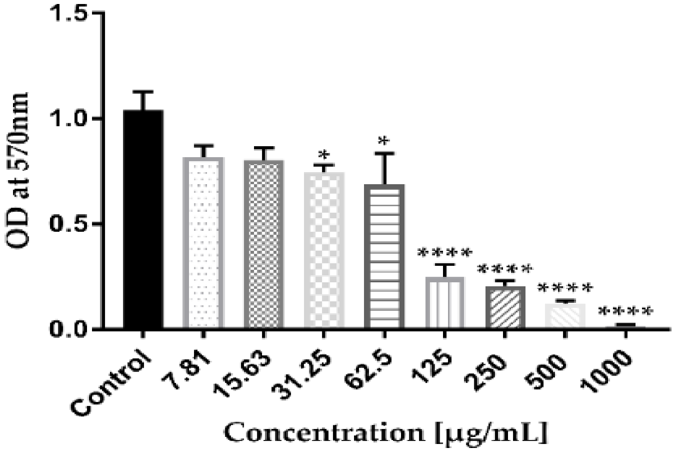

Supplementary Figure S2. Eradication and inhibition of mixed biofilm growth of three different MDR *K. pneumoniae* strains. Results are the average of three independent experiments ± STDs. Asterisks indicate statistical significance (*p =0.010, ****p < 0.0001) analysed by one-way ANOVA test with Dunnett's multiple comparison test.

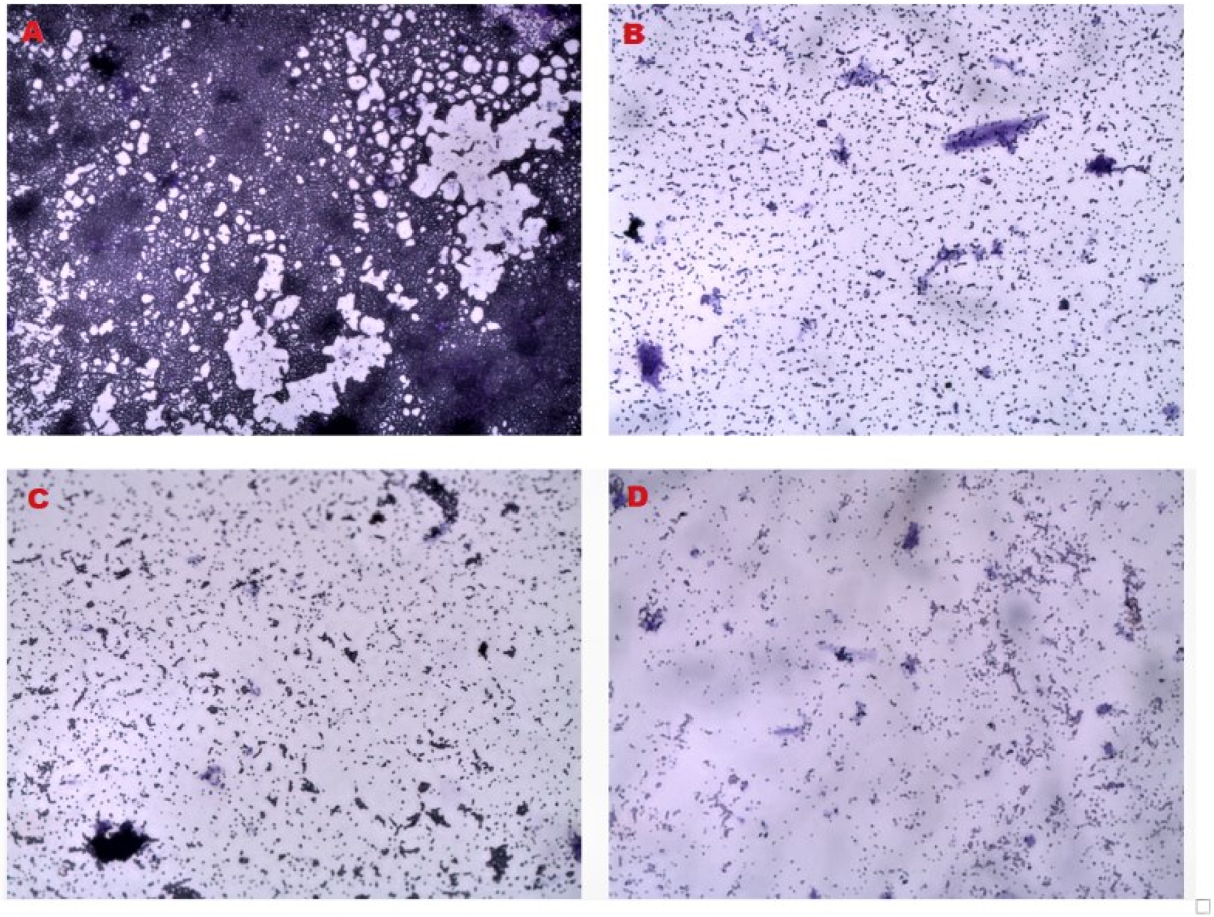

Supplementary Figure S3. Light microscopy of matured biofilms formed on glass surfaces by representative *K. pneumonie* KP ATCC-BAA2473 strain formed and disrupted after AWME3 treatment (40-x magnification). (A) Growth control without treatment; (B, C, D) Treated bacteria with AWME3 at concentrations 0.5 MIC (0.25 mg/mL), MIC (0.5 mg/mL), and 2 MIC (1 mg/mL), respectively.

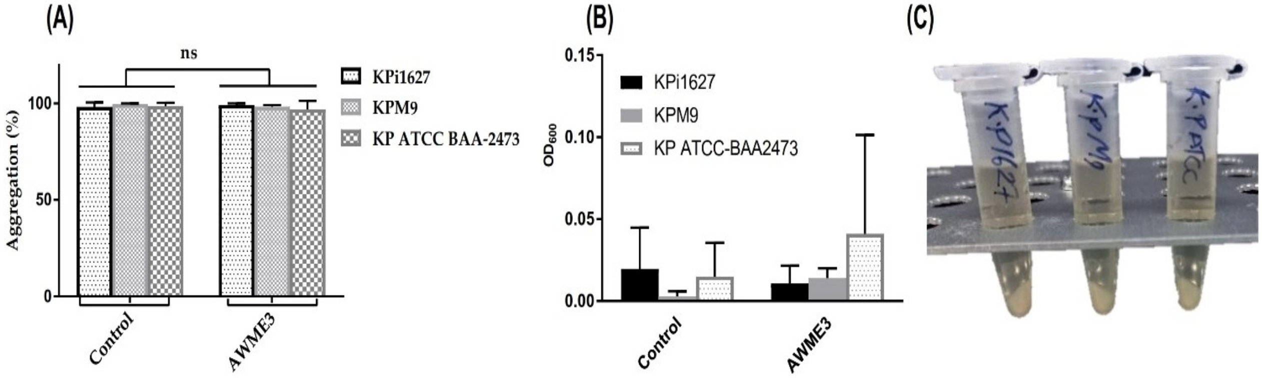

Supplementary Figure S4. Autoggregation, mucoviscosity, and precipitation assays of *K. pneumoniae* strains. KPi1627, KPM9, and KP ATCC BAA-2473 strains were cultured in LB broth overnight at 37 °C. Then, bacterial cells treated with 0.5 MIC (0.125 mg/mL) of AWME3 did not show any turbidity with low optical density of bacterial supernatant, designating the absence of mucoidity (A, B). After centrifugation

for 10 minutes at low speed (1000 ×g) the high precipitation of treated and untreated strains with dense pellets were formed (C).

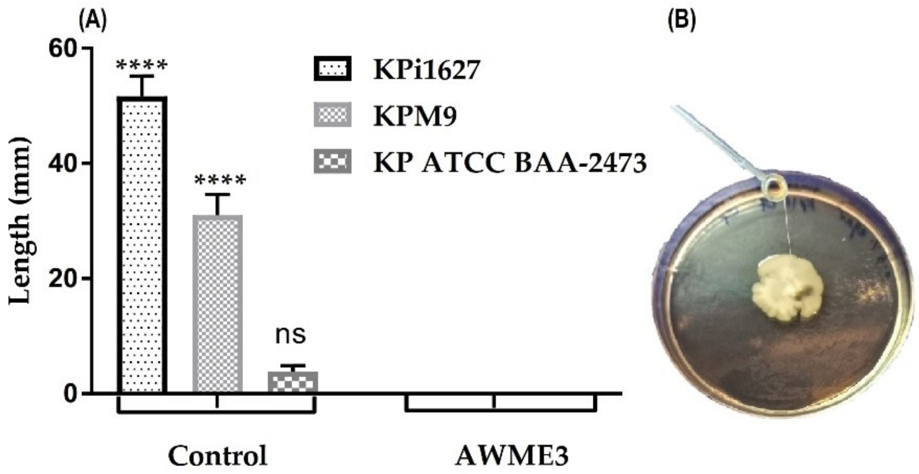

Supplementary Figure S5. String test for *K. pneumoniae* strains. Bacterial strains were cultured on LB agar plates, incubated for 72 h, and then a sterilized loop was used to stretch the bacterial colony grown on the Petri dishes agar. All strains exposed to 0.5 MIC (0.125 mg/mL) of AWME3 and all treated colonies were negative for string test (A) compared to the control group (B). The results are expressed as mean ± STD of three independent experiments. A two-way ANOVA test and Tukey's multiple comparisons test was performed to determine the statistical significance (****p≤0.0001).

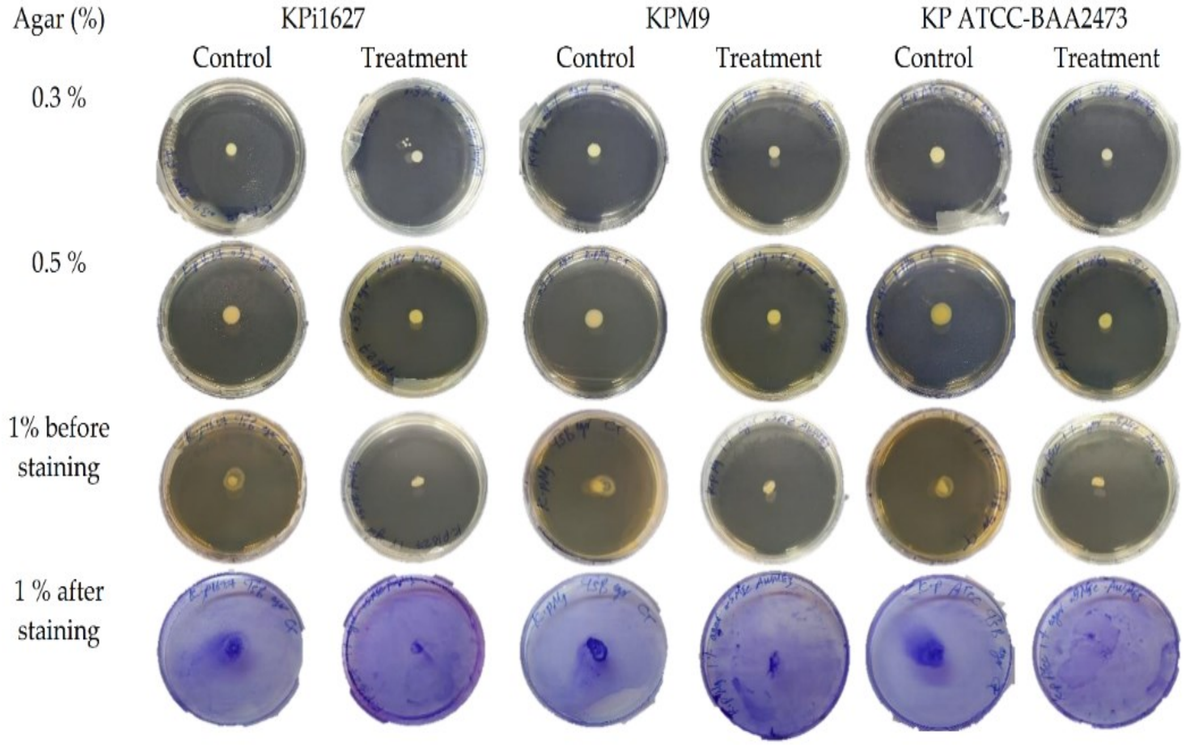

Supplementary Figure S6. Swimming (0.3% agar), swarming (0.5% agar), and twitching rudimentary motility (1% agar) of MDR *K. pneumoniae* strains: clinical isolate KPi1627, environmental isolate KPM9, and laboratorial strain KP ATCC-2473 in the presence and absence of AWME3 at 0.5MIC (0.125 mg/mL).

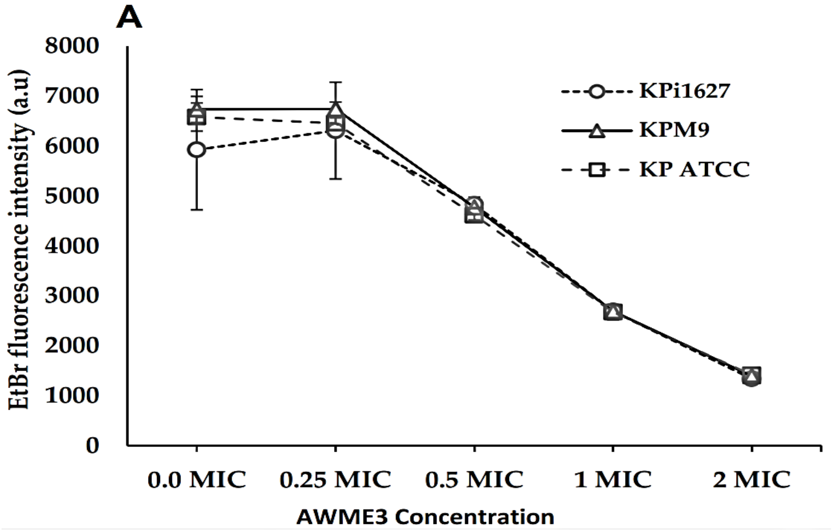

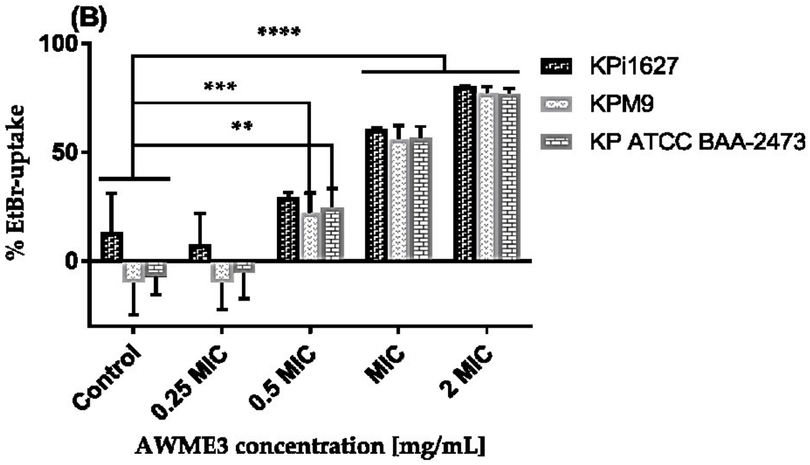

Supplementary Figure S7. Membrane disruption as measured by ethidium bromide uptake by *K. pneumoniae* KPi1627, *K. pneumoniae* KPM9, and *K. pneumoniae* KPATCC-BAA2473 strains. All strains were treated with 0.25 MIC (0.0625 mg/mL), 0.5 MIC (0.125 mg/mL), MIC (0.25 mg/mL), and 2 MIC (0.5 mg/mL) of AWME3 for 8 h. Membrane disruption was determined by measuring the fluorescence intensity values of EtBr (A); the absorbance of EtBr uptake in triplicate (B). All data are the average ± STD of three independent experiments. A tow-way ANOVA test and Dunnett's multiple comparisons test was performed to determine the statistical significance (**$p=0.001$, ***$p\leq0.0009$, ****$p\leq0.0001$).

Supplementary Tables

Supplementary Table S1. Effect of AWME3 on the aggregation of *K. pneumoniae* strains.

**KPi1627**		**KPM9**		**KP ATCC**	
Control	Treated	Control	Treated	Control	Treated
98.2±2.3	99.9±0.9	99.7±0.28	98.4±0.74	98.6±1.87	97.05±4.3

Supplementary Table S2. Effect of 0.5 MIC of AWME3 influence on the hypermucovicosity of *K. pneumoniae* strains.

**Strain**<br>**String Length (mm)**	**KPi1627**	**KPM9**	**KP ATCC-BAA2473**
Control	51.7±3.5	31±3.6	3.83±1
Treated	0.0	0.0	0.0

Supplementary Table S3. Swimming, swarming, and twitching rudimentary motility in MDR *K. pneumoniae* strains, clinical isolate (KPi1627), environmental isolate (KPM9), and laboratorial isolate (KP ATCC BAA-2473) in the presence and absence of AWME3 at 0.5MIC.

**Agar (%)**	**KPi1627**		**KPM9**		**KP ATCC-BAA-2473**	
	Control	Treatment	Control	Treatment	Control	Treatment
0.3 %	6±0.5	5.23±0.25	7.5±0.5	5.57±0.4	8.9±0.36	5.3±0.35
0.5 %	8.5±0.5	7.43±0.4	8.83±0.76	7.1±0.36	9.43±0.4	7.4±0.4
1%	8.5±0.5	4.23±0.25	9.3±0.61	4.5±0.5	10.5±0.5	4.47±0.25

Supplementary Table S4. Percent of adherence (hydrophobicity) of three *K. pneumoniae* strains treated with 0.5 MIC of AWME3.

**Hydrocarbons**	**KPi1627**		**KPM9**		**KP ATCC BAA-2473**	
	Control	Treated	Control	Treated	Control	Treated
Chloroform	36.31±2.84	64.9±6.26	44.85±2.03	38.3±2.37	35.99±1.37	44.28±1.7
Hexane	2.42±3.25	10.6±2.9	6.62±5.9	6.62±2.96	1.09±2.7	24.72±4.75
Ethyl acetate	32.46±13.9	31.05±5.9	31.85±13.97	34.87±12.36	23.99±6.42	44.6±4.25
Toluene	31.19±1.06	33.76±4.3	19.42±4.4	37.55±2.86	41.84±12.28	62.2±1.1

References

Al-Marri, T., Al-Marri, A., Al-Zanbaqi, R., Ajmi, A. Al, and Fayez, M. (2021). Multidrug resistance, biofilm formation, and virulence genes of *Escherichia coli* from backyard poultry farms. *Vet World* 14, 2869–2877. doi:10.14202/vetworld.2021.

Bellon-Fontaine, M. N., Rault, J., and Van Oss, C. J. (1996). Microbial adhesion to solvents: A novel method to determine the electron-donor/electron-acceptor or Lewis acid-base properties of

microbial cells. *Colloids Surfaces B Biointerfaces* 7, 47–53. doi:10.1016/0927-7765(96)01272-6.

Chiarelli, A., Cabanel, N., Rosinski-Chupin, I., Zongo, P. D., Naas, T., Bonnin, R. A., et al. (2020). Diversity of mucoid to non-mucoid switch among carbapenemase-producing *Klebsiella pneumoniae*. *BMC Microbiol* 20, 1–14. doi:10.1186/s12866-020-02007-y.

Hobby, C. R., Herndon, J. L., Morrow, C. A., Peters, R. E., Symes, S. J. K., and Giles, D. K. (2019). Exogenous fatty acids alter phospholipid composition, membrane permeability, capacity for biofilm formation, and antimicrobial peptide susceptibility in *Klebsiella pneumoniae*. *Microbiologyopen* 8, 1–11. doi:10.1002/mbo3.635.

Lara, H. H., and Lopez-Ribot, J. L. (2020). Inhibition of mixed biofilms of *Candida albicans* and methicillin-resistant *Staphylococcus aureus* by positively charged silver nanoparticles and functionalized silicone elastomers. *Pathogens* 9, 1–14. doi:10.3390/pathogens9100784.

Lin, Z. W., Zheng, J. X., Bai, B., Xu, G. J., Lin, F. J., Chen, Z., et al. (2020). Characteristics of hypervirulent *Klebsiella pneumoniae*: Does low expression of *rmp*A contribute to the absence of hypervirulence? *Front Microbiol* 11, 1–11. doi:10.3389/fmicb.2020.00436.

Mondal, A., Venkataramaiah, M., Rajamohan, G., and Srinivasan, V. B. (2016). Occurrence of diverse antimicrobial resistance determinants in genetically unrelated biocide tolerant *Klebsiella pneumoniae*. *PLoS One* 11, 1–24. doi:10.1371/journal.pone.0166730.

Ramos-Vivas, J., Chapartegui-González, I., Fernández-Martínez, M., González-Rico, C., Fortún, J., Escudero, R., et al. (2019). Biofilm formation by multidrug resistant *Enterobacteriaceae* strains isolated from solid organ transplant recipients. *Sci Rep* 9, 1–10. doi:10.1038/s41598-019-45060-y.

Sateriale, D., Imperatore, R., Colicchio, R., Pagliuca, C., Varricchio, E., Volpe, M. G., et al. (2020). Phytocompounds vs. dental plaque bacteria: In vitro effects of myrtle and pomegranate polyphenolic extracts against single-species and multispecies oral biofilms. *Front Microbiol* 11, 1–15. doi:10.3389/fmicb.2020.592265.

Seukep, A. J., Fan, M., Sarker, S. D., Kuete, V., and Guo, M. Q. (2020). *Plukenetia huayllabambana* fruits: Analysis of bioactive compounds, antibacterial activity and relative action mechanisms. *Plants* 9, 1–14. doi:10.3390/plants9091111.

Xu, Q., Yang, X., Chan, E. W. C., and Chen, S. (2021). The hypermucoviscosity of hypervirulent *K. pneumoniae* confers the ability to evade neutrophil-mediated phagocytosis. *Virulence* 12, 2050–2059. doi:10.1080/21505594.2021.1960101.
